# Genotype is associated with left ventricular reverse remodelling and early events in recent‐onset dilated cardiomyopathy

**DOI:** 10.1002/ehf2.15009

**Published:** 2024-08-11

**Authors:** Milos Kubanek, Jana Binova, Lenka Piherova, Alice Krebsova, Martin Kotrc, Hana Hartmannova, Katerina Hodanova, Dita Musalkova, Viktor Stranecky, Tomas Palecek, Anna Chaloupka, Ilga Grochova, Jan Krejci, Jana Petrkova, Vojtech Melenovsky, Stanislav Kmoch, Josef Kautzner

**Affiliations:** ^1^ Department of Cardiology Institute for Clinical and Experimental Medicine Prague Czech Republic; ^2^ European Reference Network for Rare, Low Prevalence and Complex Diseases of the Heart, ERN GUARD‐Heart, IKEM Prague Czech Republic; ^3^ Institute of Physiology, First Faculty of Medicine Charles University Prague Czech Republic; ^4^ Department of Pediatrics and Adolescent Medicine, First Faculty of Medicine, Research Unit for Rare Diseases Charles University Prague Czech Republic; ^5^ Department of Cardiovascular Medicine, Second Department of Medicine, First Faculty of Medicine Charles University and General University Hospital Prague Czech Republic; ^6^ First Internal Clinic of Cardio‐Angiology St. Anne's University Hospital and Medical School of Masaryk University Brno Czech Republic; ^7^ Department of Internal Medicine I – Cardiology University Hospital Olomouc Olomouc Czech Republic; ^8^ Department of Pathological Physiology, Faculty of Medicine and Dentistry Palacky University Olomouc Olomouc Czech Republic

**Keywords:** Genetics, Left ventricular reverse remodelling, Prognosis, Recent‐onset dilated cardiomyopathy, Whole‐exome sequencing

## Abstract

**Aims:**

Recent‐onset dilated cardiomyopathy (RODCM) is characterized by heterogeneous aetiology and diverse clinical outcomes, with scarce data on genotype–phenotype correlates. Our aim was to correlate individual RODCM genotypes with left ventricular reverse remodelling (LVRR) and clinical outcomes.

**Methods and results:**

In this prospective study, a total of 386 Czech RODCM patients with symptom duration ≤6 months underwent genetic counselling and whole‐exome sequencing (WES). The presence of pathogenic (class 5) or likely pathogenic (class 4) variants in a set of 72 cardiomyopathy‐related genes was correlated with the occurrence of all‐cause death, heart transplantation, or implantation of a ventricular assist device (primary outcome) and/or ventricular arrhythmia event (secondary outcome). LVRR was defined as an improvement of left ventricular ejection fraction to >50% or ≥10% absolute increase, with a left ventricular end‐diastolic diameter ≤33 mm/m^2^ or ≥10% relative decrease. Median follow‐up was 41 months. RODCM was familial in 98 (25%) individuals. Class 4–5 variants of interest (VOIs) were identified in 125 (32%) cases, with 69 (18%) having a single titin‐truncating variant (*TTNtv*) and 56 (14%) having non‐titin (non‐*TTN*) VOIs. The presence of class 4–5 non‐*TTN* VOIs, but not of *TTNtv*, heralded a lower probability of 12‐month LVRR and proved to be an independent baseline predictor both of the primary and the secondary outcome. The negative result of genetic testing was a strong protective baseline variable against occurrence of life‐threatening ventricular arrhythmias. Detection of class 4–5 VOIs in genes coding nuclear envelope proteins was another independent predictor of both study outcomes at baseline and also of life‐threatening ventricular arrhythmias after 12 months. Class 4–5 VOIs of genes coding cytoskeleton were associated with an increased risk of life‐threatening ventricular arrhythmias after baseline assessment. A positive family history of dilated cardiomyopathy alone only related to a lower probability of LVRR at 12 months and at the final follow‐up.

**Conclusions:**

RODCM patients harbouring class 4–5 non‐*TTN* VOIs are at higher risk of progressive heart failure and life‐threatening ventricular arrhythmias. Genotyping may improve their early risk stratification at baseline assessment.

## Graphical abstract

In this prospective prognostic study, a total of 386 Czech patients with recent‐onset dilated cardiomyopathy (RODCM) underwent genetic counselling and whole‐exome sequencing (WES). Carriers of pathogenic/likely pathogenic non‐titin variants of interest (VOIs) (14%) had a lower probability of 12‐month left ventricular reverse remodelling (LVRR). They had an increased risk of death and progressive heart failure and also a burden of life‐threatening ventricular arrhythmias in comparison with genotype‐negative individuals. Genetic testing therefore could improve early risk stratification of RODCM cases.

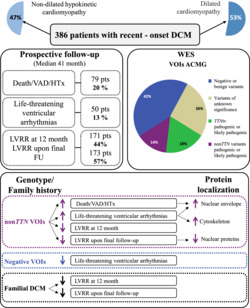

## Introduction

The heterogeneous aetiology of dilated cardiomyopathy (DCM), which consists of genetic, inflammatory, toxic, and metabolic causes, complicates accurate diagnostic and prognostic classification of the disease.^1^ This is most evident in individuals with recent‐onset dilated cardiomyopathy (RODCM), who develop a broad spectrum of outcomes ranging from end‐stage heart failure or sudden cardiac death to a more favourable outcome of left ventricular reverse remodelling (LVRR).^2^, ^3^ LVRR is characterized by improvements in left ventricular (LV) systolic function along with a substantial reduction in ventricular volumes.^2^ The reversibility of heart failure is more likely in arrhythmic, alcoholic, or inflammatory aetiologies than in idiopathic or genetic diseases.^3^, ^4^ In addition to heart failure progression, patient survival may be compromised by life‐threatening ventricular arrhythmias, which predominate in ‘arrhythmic phenotypes’.^5^, ^6^, ^7^ Mutations in lamin A/C,^8^, ^9^ phospholamban,^10^ filamin C,^11^, ^12^ RNA‐binding motif protein 20,^13^ and BCL2‐associated athanogene 3^14^ are associated with a poor prognosis due to progressive heart failure and/or arrhythmic events. Genetic assessment of RODCM patients thus could be helpful for early decision‐making regarding timing of primary preventive implantation of cardioverter‐defibrillators (ICD) and heart transplant enlistment.

Currently, more than 50 genes are considered disease‐related, with causative variants identified in approximately 20% to 50% of all DCM cases.^15^, ^16^, ^17^ Titin (*TTN*) is the most commonly affected gene, with titin‐truncating variants (*TTNtv*) responsible for 19 to 25% of familial, and 11 to 18% of sporadic cases.^18^ Data on the prognostic implications of genetic testing in DCM and RODCM patients are scarce and mainly comprise retrospective analyses^19^, ^20^ and registry reports^21^, ^22^, ^23^, ^24^ of DCM patients; there are no studies that exclusively focus on RODCM. Importantly, a recent report from the Swedish Heart Failure Registry revealed a better prognosis of RODCM in comparison with chronic DCM.^25^ RODCM thus represents a different disease than chronic DCM also from a prognostic point of view.

The aim of this study was to assess the genetic architecture of RODCM in a real‐life cohort of patients referred to tertiary hospitals. Whole‐exome sequencing (WES) was used as the initial genetic test to determine genetic heterogeneity of DCM. The research question was whether the individual genetic background of RODCM would both correlate with LVRR and predict clinical outcomes of death and progression to end‐stage heart failure and/or life‐threatening ventricular arrhythmias. As the initial studies suggested a better prognosis^20^ and/or a higher probability of LVRR among carriers of *TTNtv* than in laminopathy^20^ or in carriers of non‐titin (non‐*TTN*) variants,^23^ we analysed outcomes of *TTNtv* and non‐*TTN* variant carriers separately.

## Methods

### Study population

The study conformed to the ethical principles of the Declaration of Helsinki and was approved by the local institutional review boards of the participating institutions. All patients provided their written informed consent. This was a three‐centre prospective observational study of unrelated adult RODCM patients (age ≥18 years) with symptoms lasting no longer than 6 months upon initial assessment. RODCM was defined as left ventricular or biventricular systolic dysfunction (defined as a left ventricular ejection fraction <45%) with or without left ventricular dilatation unexplained by abnormal loading conditions or coronary artery disease^26^ (Data S1). Exclusion criteria comprised biopsy‐proven acute myocarditis, treatment of any form of myocarditis with specific antibiotic, antiviral or immunosuppressive therapy, suspected arrhythmia‐induced, toxic, metabolic or endocrine aetiology, and scheduled cardiac resynchronization therapy within 12 months (Data S1). The subjects were prospectively enrolled from heart failure centres of three major tertiary hospitals from January 2005 to June 2017.

### Definitions

RODCM was considered familial in either of the following cases: (i) DCM diagnosis of at least two genetically related first‐degree relatives; (ii) premature sudden cardiac death or heart failure in a first‐degree relative at <35 years of age.^22^ Treated ventricular tachyarrhythmia included (i) fast sustained ventricular tachycardia, (ii) ventricular fibrillation requiring cardioversion or (iii) appropriate intervention of an ICD (shock or anti‐tachycardia pacing). LVRR was defined as an improvement in LV ejection fraction to >50% or ≥10% absolute increase, with the follow‐up left‐ventricular end‐diastolic diameter ≤33 mm/m^2^ or a relative decrease ≥10%.^23^

### Study protocol

Initial patient assessment included analysis of family history over three generations, physical examination, electrocardiography, echocardiography, routine blood tests, and collection of 5 mL peripheral venous blood for genetic testing. All patients received standard heart failure management according to the guidelines.^27^ This consisted of repeated clinical examination, electrocardiography, echocardiography and routine blood tests at 12 months to assess cardiac remodelling, followed by a request to continue follow‐up every 12 months thereafter. Echocardiography was performed in a standard manner, with the LV ejection fraction assessed using Simpson's biplane method.^28^, ^29^ In participants with implantable pacemakers or ICDs, device function, arrhythmia detection, and applied treatment were checked every 6 months.

### Study outcomes

**Figure 1:**
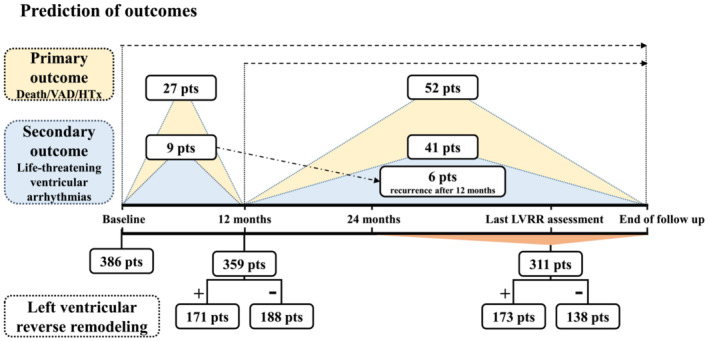
Timeline illustrates prediction of the study outcomes both from baseline and 12 months of follow‐up.

The primary study outcome was pre‐specified as the first event of all‐cause death or the necessity of heart transplantation or implantation of a ventricular assist device. Secondary study outcomes included (i) the event of life‐threatening ventricular arrhythmia ‐ the first event of sudden cardiac death (defined in Data S1), resuscitated cardiac arrest or treated ventricular tachyarrhythmias; (ii) LVRR at 12 months; (iii) LVRR upon final follow‐up based on comparison between the baseline and the last echocardiography available after ≥2 years of follow‐up. *Figure* *1* illustrates prediction of study outcomes from baseline and 12 months of follow‐up. All available clinical data and events were collected up until 30 July 2018. The vital status of each patient was ascertained through a health insurance registry up until the same date. Where possible, families and general practitioners were contacted in cases of out‐of‐hospital deaths. Both the provision of clinical care and outcome adjudication were blinded to genotype.

### Genetic analysis

WES was performed in a standard manner.^30^, ^31^ For technical details, see Data S1. For the purpose of this study, 72 DCM‐related genes were identified based on the keyword ‘dilated cardiomyopathy’, with each gene found in at least two of the following six databases: OMIM, ClinVar, HPO, Orphanet, HGMD and GeneCards (Table S1). Although the ClinGen initiative identified just 19 genes with a moderate or definitive evidence for involvement in DCM, this approach excluded genes associated with syndromic DCM and genes with overlapping phenotype.^32^ In addition, the ClinGen curation omitted also rare and extremely rare genetic causes of DCM. We considered maintaining the inclusion of these genes in our set of DCM‐related genes clinically important because we aimed to reveal a broad genetic background and extracardiac features may be subtle and clinically unrecognized.^33^

Variants in DCM genes were only considered putative disease‐causing mutations in cases where (i) population frequencies in publicly available genotype data from subjects of European origin obtained from The Genome Aggregation Database (GnomAD)^34^ and ethnically matched population controls maintained by the Czech National Center for Medical Genomics (*n* = 1055) were ≤0.05% and (ii) conservation scores (GERP) across different species exceeded 4.

Identified variants fulfilling the selected criteria were divided according to the standard variant classification of the American College of Medical Genetics and Genomics (ACMG)^30^, ^31^ into three groups: (1) class 3—variants of uncertain significance (VUS); (2) class 4—likely pathogenic variants; (3) class 5—pathogenic variants. To stay on the safe side, we included just class 4**–**5 variants in our analysis as putative disease‐causing variants of interest (VOIs). A separate group represented individuals with VUS, other results of genetic testing were classified as negative. As explained in the introduction, we analysed outcomes in carriers of *TTNtv* and non‐*TTN* VOIs separately.

All subjects underwent pre‐test genetic counselling. Patients with familial RODCM or identified as having a class 4–5 mutation or VUS were invited by a letter to attend also a post‐test genetic counselling session performed by a clinical geneticist. In total, 228 (59%) of 386 patients underwent post‐test genetic counselling, including 73 individuals with a positive family history of DCM and 155 individuals with positive genetic findings for class 4–5 VOIs and some VUS. First‐degree relatives were invited to undergo clinical screening and gene‐specific genetic testing according to guidelines using the Sanger sequencing method to study segregation in families.^35^ Familial segregation of variants was completed in 113 families.

### Statistical analysis

Details of the statistical analysis are provided in Data S1.

## Results

A total of 386 consecutive unrelated RODCM patients, Caucasians of the Czech origin, were enrolled between January 2005 and June 2017 (*Table* *1*). Their cardiac phenotype was consistent with dilated cardiomyopathy in 205 subjects (53%) and non‐dilated left ventricular cardiomyopathy (non‐dilated hypokinetic cardiomyopathy) in 181 subjects (47%).^26^ Endomyocardial biopsy was performed in 167 (43%) patients based on a clinical indication and excluded acute myocarditis in all cases according to Dallas criteria.^36^ Details of the pharmacological and device management are shown in *Table* *1* and Data S1. During the median follow‐up of 41 months (25–64), the initial primary outcome event was all‐cause death in 32 (9%) patients (Data S1), heart transplantation in 24 (6%) patients, and implantation of a long‐term ventricular assist device in 23 (6%) patients (*Figure* *1*, Central Illustration). The incidence of the primary outcome was 4.93 events per 100 patient‐years. The secondary end‐point of life‐threatening ventricular arrhythmias occurred in 50 (13%) individuals, with sudden cardiac death in 8 (2%) cases, successfully resuscitated cardiac arrest due to ventricular fibrillation in one (0.3%) case, and adequate ICD intervention due to fast ventricular tachyarrhythmia in 41 (11%) patients. The incidence of this secondary outcome was 3.31 events per 100 patient‐years.

**Table 1:** Baseline characteristics the study group

	Baseline characteristics (*n* = 386)	Baseline data, finished 12‐month follow‐up (*n* = 359)	Baseline data, HTx, LVAD or death before 12 months (*n* = 27)	Baseline data, LVRR present at 12 months (*n* = 171)	Baseline data, LVRR absent at 12 months (*n* = 188)
Age (years)	44 ± 12	45 ± 12	32 ± 9***	45 ± 11	46 ± 13
Males	277 (72%)	257 (72%)	20 (74%)	116 (68%)	141 (75%)
Familial DCM	98 (25%)	90 (25%)	8 (30%)	26 (15%)	64 (34%)***
Diabetes mellitus	37 (10%)	37 (10%)	0	15 (9%)	22 (12%)
Arterial hypertension	103 (27%)	102 (28%)	1 (4%)**	53 (31%)	49 (26%)
Asthma bronchiale	25 (6%)	22 (6%)	3 (11%)	11 (6%)	11 (6%)
History of persistent atrial fibrillation	60 (16%)	55 (15%)	5 (18%)	18 (11%)	37 (20%)*
Viral prodroms	147 (38%)	135 (38%)	12 (44%)	73 (43%)	62 (33%)
Decompensated HF at admission	148 (38%)	132 (37%)	16 (60%)*	71 (42%)	61 (32%)
Manifestation by sustained ventricular arrhythmia	11 (3%)	11 (3%)	0	3 (1%)	8 (2%)
NYHA class					
I	30 (8%)	30 (9%)	0***	12 (7%)	18 (10%)
II	202 (52%)	199 (55%)	3 (11%)	93 (54%)	106 (56%)
III	121 (31%)	108 (30%)	13 (48%)	51 (30%)	57 (30%)
IV	33 (9%)	22 (6%)	11 (41%)	15 (9%)	7 (4%)
ACEI or ARB	308 (80%)	294 (82%)	14 (52%)**	144 (84%)	150 (80%)
ACEI/ARB ≥ 50% of recommended dose (%)	142 (36)	140 (39)	2 (7)**	62 (36)	78 (41)
Beta‐blockers	326 (85%)	313 (87%)	13 (48%)***	149 (88%)	164 (87%)
Beta‐blockers ≥ 50% of recommended dose (%)	109 (28)	107 (30)	2 (7)*	56 (33)	52 (27)
Aldosteron receptor blockers	272 (81%)	250 (70%)	22 (81%)	127 (75%)	123 (66%)
Furosemide	310 (80%)	286 (80%)	24 (89%)	142 (83%)	144 (77%)
Furosemide ≥ 40 mg/day (%)	235 (61)	212 (59)	23 (85)**	106 (62)	106 (56)
Digoxin	31 (8%)	28 (8%)	3 (11%)	11 (6%)	17 (9%)
Intravenous diuretics	70 (18%)	53 (15%)	17 (63%)***	27 (16%)	26 (14%)
Inotropes	39 (10%)	21 (6%)	18 (67%)***	11 (6%)	9 (5%)
BMI (kg/m^2^)	28 ± 9	28 ± 9	26 ± 8*	27 ± 12	27 ± 9
Systolic BP (mmHg)	119 ± 18	120 ± 18	105 ± 12***	120 ± 18	120 ± 19
Diastolic BP (mmHg)	76 ± 12	76 ± 13	70 ± 10**	78 ± 13	76 ± 12
Heart rate (b.p.m.)	84 ± 17	83 ± 17	97 ± 19***	86 ± 18	81 ± 16*
Sinus rhythm	372 (97%)	348 (97%)	24 (89%)	167 (98%)	181 (97%)
QRS duration (ms)	106 ± 27	107 ± 27	104 ± 17	102 ± 23	111 ± 30**
Complete LBBB	71 (18%)	69 (19%)	2 (7%)	28 (16%)	41 (22%)
LVEDD (mm)	67 ± 7	67 ± 7	71 ± 6**	66 ± 7	67 ± 7
LVEDD (mm/m^2^)	33 ± 5	33 ± 5	38 ± 7***	33 ± 5	34 ± 5*
Interventricular septum (mm)	9 ± 2	9 ± 2	8 ± 1	9 ± 2	9 ± 2
Posterior wall (mm)	9 ± 1	9 ± 1	8 ± 1	9 ± 1	9 ± 1
LVEF (%)	24 ± 8	25 ± 8	18 ± 4***	23 ± 7	26 ± 7***
Restrictive mitral inflow pattern (*n* = 292)	120 (41%)	109 (39%)	11 (85%)***	57 (41%)	51 (36%)
E/E′ ratio (*n* = 319)	13.4 ± 6.3	13.3 ± 6.3	15.6 ± 6.0	13.2 ± 6.4	13.4 ± 6.3
Left atrium short axis (mm)	46 ± 7	46 ± 7	47 ± 5	46 ± 7	46 ± 6
Left atrium long axis (mm) (*n* = 271)	58 ± 10	58 ± 10	63 ± 10*	58 ± 11	58 ± 8
LAVI (mL/m^2^) (*n* = 241)	48 ± 17	47 ± 17	66 ± 17***	47 ± 16	47 ± 17
Mitral regurgitation ≥ moderate	113 (29%)	94 (26%)	19 (70%)***	37 (22%)	57 (30%)**
RVD1 (mm) (*n* = 281)	37 ± 7	37 ± 7	43 ± 7***	38 ± 7	36 ± 6*
Tricuspid annulus Sm (*n* = 328)	10.1 ± 3.6	10.1 ± 3.1	10.1 ± 8.4	9.7 ± 2.6	10.5 ± 3.4
TAPSE (mm) (*n* = 321)	18 ± 4	18 ± 3	14 ± 3***	18 ± 4	19 ± 3
Tricuspid regurgitation ≥ moderate (*n* = 356)	41 (11%)	36 (10%)	5 (16)**	14 (8%)	22 (12%)
Sodium (mmol/L)	139.0 ± 3.4	139.4 ± 1.1	135.2 ± 4.5***	139.4 ± 3.2	139.4 ± 3.1
Creatinine (μmol/L)	90.7 ± 22.2	90.4 ± 22.2	93.9 ± 21.3	90.6 ± 23.5	90.4 ± 21.1
Estimated GFR (mL/min)	111 ± 38	111 ± 38	111 ± 42	113 ± 39	109 ± 37
BNP (ng/L) (*n* = 261)	316 (123–840)	278 (109–674)	1362 (787–2039)***	277 (107–680)	279 (113–661)
NT‐proBNP (ng/L) (*n* = 84)	1664 (752–3232)	1664 (752–3232)	‐	1689 (753–3345)	1386 (733–3007)
BNP/NT‐proBNP quartile					
1st	85 (25%)	85 (26%)	0***	45 (27%)	40 (25%)
2nd	88 (25%)	88 (27%)	0	40 (25%)	48 (30%)
3rd	86 (25%)	79 (24%)	7 (30%)	42 (26%)	37 (23%)
4th (*n* = 345)	86 (25%)	70 (22%)	16 (70%)	36 (22%)	34 (21%)
hs‐cTNT (ng/L) (*n* = 187)	14.5 (5.0–30.0)	14.8 (9.0–30.0)	18.5 (10.0–42.5)	14.8 (8.6–29)	14.5 (9.3–30)
Troponin I (μg/L) (*n* = 86)	0.03 (0.00–0.07)	0.03 (0.00–0.06)	0.06 (0.00–0.35)	0.03 (0.00–0.05)	0.03 (0.0075–0.12)
Troponin I > 0.03 or hs‐cTNT >13.5 ng/L (*n* = 243)	116 (48%)	109 (47%)	7 (54%)	61 (48%)	48 (46%)

The second and the third column illustrate differences between patients who finished the 12‐month follow‐up and those with the primary end‐point (heart transplantation, implantation of left ventricular assist device, or death) before 12 months. The fourth and fifth column compare baseline data of individuals with and without left ventricular reverse remodelling at 12 months.

ACEI, angiotensin converting enzyme inhibitors; ARB, angiotensin receptor blockers; BMI, body mass index; BNP, B‐type natriuretic peptide; BP, blood pressure; DCM, dilated cardiomyopathy; GFR, glomerular filtration rate; hs‐cTNT, high sensitivity cardiac troponin T; LBBB, left bundle branch block; LVEDD, left ventricular end‐diastolic dimension; LVEF, left ventricular ejection fraction; LAVI, left atrial volume index; NT‐proBNP, N‐terminal pro‐B‐type natriuretic peptide; RVD1, basal right ventricular diameter; TAPSE, tricuspid annular systolic plane excursion.

* *P* < 0.05.

** *P* < 0.01.

*** *P* < 0.001.

### Genetic background of recent‐onset dilated cardiomyopathy

**Figure 2:**
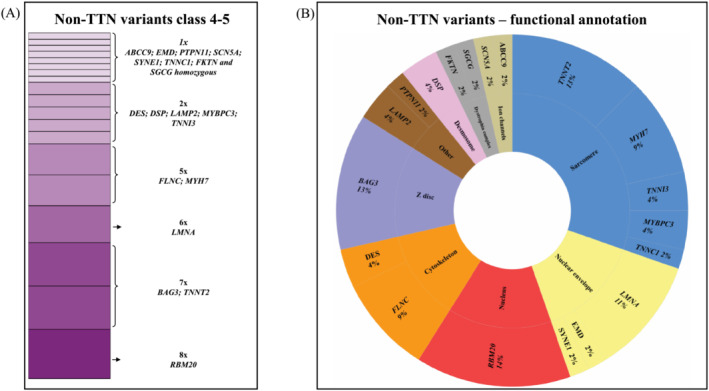
(A) Spectrum of affected genes among class 4–5 non‐titin (non‐*TTN*) variants detected in the study group. Their functional annotation is described in panel (B).

In the pre‐defined set of 72 DCM genes, WES detected a single class 4–5 titin truncating variant (*TTNtv*) in 69 pts (18%), a single class 4–5 non‐titin variant (non‐*TTN*) in 56 pts (14%), only VUS in 103 pts (27%) and a negative result in 158 pts (41%). Just one subject had a combination of class 4–5 *TTNtv* with class 4–5 non‐*TTN* variant. He was included in the non‐*TTN* group in further analyses. *Figure* *2* *A* illustrates spectrum of affected genes among non‐*TTN* variants. *Figure* *2* *B* reveals their functional annotation. Class 4–5 non‐*TTN* variants affected most frequently sarcomeric genes (32%), genes coding nuclear envelope (predominantly *LMNA*) (15%), nuclear components (*RBM20*) (14%), cytoskeleton (*FLNC* and *DES*) (13%) and Z‐disk (*BAG3*) (13%). The remaining functional groups were less frequent. The expected mode of inheritance among most subjects with a class 4–5 variant was autosomal dominant (94.4%), with autosomal‐recessive (*SGCD*, *FKTN*, *ABCC9*, and *SYNE1*) and X‐linked (twice *LAMP2* and *EMD*) inheritance accounting for 3.2% and 2.4% of cases, respectively. All individual class 4–5 non‐*TTN* and *TTNtv* are listed in Data S2.

A positive family history of DCM was ascertained in 98 (25%) patients. Among familial RODCM, 33% of cases had class 4–5 *TTNtv*, 29% class 4–5 non‐*TTN* variants, 13% VUS, and 25% a negative genotype. In contrast, individuals with sporadic RODCM had a lower representation of class 4–5 *TTNtv* (13%) and class 4–5 non‐*TTN* variants (10%) and a higher incidence of VUS (31%) and negative results (46%) than familial cases (*P* < 0.001).

### Prediction of early occurrence for the primary outcome and 12‐month left ventricular reverse remodelling

A total of 27 (7%) patients experienced the primary outcome before 12 months, related mainly to heart failure progression (1 sudden cardiac death, 2 deaths of progressive heart failure, 13 urgent heart transplants, and 11 implants of a ventricular assist device). Clinical characteristics of these patients are shown in *Table* *1*. Genetically (Table S2), patients suffering a primary event within 12 months had more frequently class 4–5 non‐*TTN* variants (37 vs. 13%, *P* < 0.01) and also class 4–5 VOIs in genes coding nuclear envelope (11 vs. 1.4%, *P* < 0.05) than the remaining subjects.

Of the 359 patients who finished the 12‐month follow‐up (*Table* *1*), 171 (48%) patients fulfilled the definition for LVRR. Compared to patients with absent 12‐month LVRR, baseline characteristics for individuals to complete LVRR revealed less frequent history of persistent atrial fibrillation, narrower QRS complexes, slightly lower LV end‐diastolic diameter index and LV ejection fraction, and less prevalent moderate‐to‐severe mitral regurgitation. In addition, individuals with 12‐month LVRR exhibited less frequent history of familial DCM (15 vs. 34%, *P* < 0.001) and had less frequently present class 4–5 non‐*TTN* VOIs (8 vs. 18%, *P* < 0.01) (Table S2, Central Illustration). Other results of genetic testing or any gene functional groups were not associated with 12‐month LVRR.

### Prediction of left ventricular reverse remodelling at the final follow‐up

LVRR at the final follow‐up occurred in 173 (56%) of 311 individuals with an available echocardiographic follow‐up ≥2 years after baseline. Comparison of the baseline and the above specified last available echocardiography was used for the assessment. The median time from the baseline to the final echocardiography was 55 months (38–71). Modest differences in clinical and para‐clinical variables between both groups at baseline are shown in Table S3. Based on genetic assessment, individuals with LVRR at the final follow‐up had less frequent history of familial DCM (18 vs. 32%, *P* < 0.01) and a lower prevalence of both class 4–5 non*‐TTN* VOIs (5 vs. 16%, *P* < 0.01) and class 4–5 VOIs in genes coding nuclear proteins (0 vs. 5%, *P* < 0.05), which affected exclusively the gene *RBM20*.

### Prediction of clinical events at baseline

**Figure 3:**
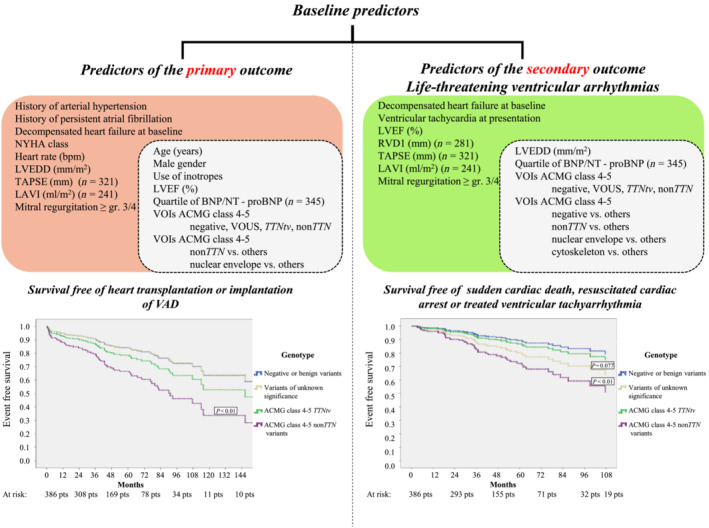
Diagrams in the upper part of the figure list variables predicting the primary and secondary outcome from baseline. The white subsets inside each of both lists summarize variables, which remained independent in most multivariate models. The Kaplan–Meier curves in the lower part show an increased occurrence of both outcomes in carriers of class 4–5 non‐titin VOIs compared to genotype‐negative individuals. Abbreviations are shown in *Table* *1*.

Based on univariate Cox regression analysis, 17 of 81 baseline variables were associated with the primary outcome (Table S4). In addition to conventional clinical and para‐clinical variables, we identified the following genetic predictors of the primary outcome: class 4–5 VOIs in non‐*TTN* genes and among gene functional groups class 4–5 VOIs in genes coding nuclear envelope. Importantly, both above mentioned genetic variables remained independent predictors of the primary outcome in multivariate models constructed of the 10 strongest predictors available for the majority of the study group (Table S4, *Figure* *3*).

Furthermore, 14 of 81 baseline variables were associated with the secondary outcome of life‐threatening ventricular arrhythmias according to univariate analysis (Table S4, *Figure* *3*). With regard to genetic predictors, the presence of class 4–5 non*‐TTN* VOIs, and detection of class 4–5 VOIs in genes coding nuclear envelope proteins and cytoskeleton were associated with an increased occurrence of this outcome. All of these genetic predictors remained independent predictors of life‐threatening ventricular arrhythmias in baseline multivariate models constructed of the eight strongest predictors available for the majority of the study group (Table S4, *Figure* *3*). Family history of DCM alone was neither associated with the primary nor the secondary outcome of life‐threatening ventricular arrhythmias.

### Prediction of clinical events at 12‐month follow‐up

We also evaluated the relationship of 12‐month variables and genetic testing results to the primary and secondary outcomes after 12 months of follow‐up. This analysis should clarify whether genetic variables improve prediction of the above‐mentioned outcomes in comparison with the latest test results including LVRR and LV ejection fraction at 12 months. For a summary of the significant variables, see Table S5. Class 4–5 VOIs in genes coding nuclear envelope was the only genetic predictor of the primary outcome after 12 months, albeit non‐significant according to multivariate analysis. Interestingly, several genetic variables predicted the secondary outcome of life‐threatening ventricular arrhythmias after 12 months at univariate analysis. These included mainly class 4–5 VOIs in non‐*TTN* genes and class 4–5 VOIs in genes coding nuclear envelope and cytoskeleton. However, just class 4–5 VOIs in genes coding nuclear envelope remained significant in multivariable models together with 12‐month LVEF or with the presence of LVRR at 12 months (Table S5).

## Discussion

This is the first prospective study to correlate genotype with LVRR and clinical outcomes in patients with recent‐onset dilated cardiomyopathy. The main findings can be summarized as follows: (1) Likely pathogenic or pathogenic variants (class 4–5 VOIs) of DCM‐related genes were identified in 32% of RODCM patients; (2) the presence of class 4–5 non*‐TTN* variant heralded a lower probability of 12‐month LVRR and proved to be an independent baseline predictor both of the primary outcome (all‐cause death/heart transplantation/implantation of a ventricular assist device) and also of the secondary outcome of life‐threatening ventricular arrhythmias; (3) the negative result of genetic testing was a strong protective variable against occurrence of life‐threatening ventricular arrhythmias when compared with other non‐genetic predictors at baseline; (4) the presence of class 4–5 VOIs in genes coding nuclear envelope proteins was another independent predictor of both study outcomes at baseline and also of life‐threatening ventricular arrhythmias after 12 months; (5) additionally, class 4–5 VOIs in genes coding cytoskeleton strongly predicted life‐threatening ventricular arrhythmias at baseline; (6) the detection of class 4–5 non‐*TTN* variants and class 4–5 VOIs in genes coding nuclear proteins was associated with a reduced occurrence of LVRR at the final follow‐up; (7) finally, a positive family history of DCM alone only related to lower probability of LVRR at 12 months and at the final follow‐up. The findings are summarized in the Central Illustration.

### Comparison with previous studies

Retrospective in design, the first prognostic studies of DCM patients reported a better survival free of heart transplantation and life‐threatening ventricular arrhythmias in genotype‐negative patients^19^ and carriers of *TTNtv* ^20^, ^21^ compared with genotype‐positive patients, mainly carriers of *LMNA* variants. A recent paper from the Familial Cardiomyopathy Registry^22^ reported results of next‐generation sequencing in 487 DCM patients with familial disease in 60% of subjects and pathogenic/likely pathogenic variants detected in 37% of cases. Variant‐positive individuals had a borderline increase in the risk of progressive heart failure and life‐threatening ventricular arrhythmias, with the highest risk in carriers of variants in desmosomal genes and lamin A/C independent of LV ejection fraction. A study from the Maastricht Cardiomyopathy Registry^23^ of 346 patients with DCM and hypokinetic non‐dilated cardiomyopathy revealed pathogenic variants in 22% of patients using a 47‐cardiomyopathy gene panel. With the exception of *TTNtv*, these variants were strongly associated with a lower rate of LVRR but not with clinical outcomes. The largest study evaluated retrospectively the prognostic value of genetic testing in 1005 DCM patients recruited from 20 Spanish centres.^24^ DCM was familial in 48% of cases and pathogenic/likely pathogenic variants were identified in 37% of subjects with predominance of non‐*TTN* variants (69%) over *TTNtv* (31%). Genotype‐positive patients had a higher incidence of end‐stage heart failure and life‐threatening ventricular arrhythmias than genotype‐negative subjects. In addition, the positive genetic testing was associated with a lower occurrence of LVRR (39.6% vs. 46.2%, *P* = 0.047)^24^; however, prevalence of LVRR ranged from 53% in carriers of *TTNtv*, to 25% in carriers of pathogenic variants of genes coding nuclear envelope or 11% in carriers of pathogenic variants of desmosomal genes. Studies of Gigli et al.^22^ and Escobar‐Lopez et al.^24^ report a very high prevalence of familial disease (48% and 60%), which was 25% in our study and 28% in the study of Verdonschot et al.^23^ This excess of familial disease may reflect a selection bias based on prevailing enrolment of patients from units for inherited heart disease. Such studies thus may not represent the real‐life spectrum of the disease in general population.

Similar to Verdonschot et al.,^23^ we found that both positive family history of DCM and presence of class 4–5 non‐*TTN* VOIs may have a deleterious effect on LVRR. Contrary to our observation, prevalence of *TTNtv* was increased among subjects with LVRR in the Maastricht and Spanish cohorts^23^, ^24^ suggesting a higher reversibility of titin‐related DCM in these study groups. The prospective assessment of LVRR is the main advantage of our study, which supports the reliability of our results. In contrast, registries and retrospective studies may be suboptimal, as they assess LVRR in variable time frames and provide results only in patients with available data.

Importantly, we confirmed among RODCM patients a strong relationship between the positive genetic test (presence of class 4–5 non*‐TTN* VOIs and/or class 4–5 VOIs in genes coding nuclear envelope proteins) and both the primary outcome (death/progressive heart failure) and the secondary outcome (life‐threatening ventricular arrhythmias) (Central Illustration, *Figure* *3*). This is in agreement with previous studies showing poor prognosis of laminopathies^21^, ^22^ and also with the latest paper of Escobar‐Lopez et al.^24^ The absent association between the genotype and the outcome in the study of Verdonschot et al.^23^ might be related to recruitment of relatively stable subjects with a low prevalence of adverse events during the median 50 months follow‐up (13 cardiovascular deaths, 3 heart transplants, no reported ventricular‐assist device, 33 life‐threatening arrhythmias). A low morbidity and mortality in this study suggest enrolment of milder cases from out‐patient settings than in our study. We showed that 7% of the study group experienced the primary outcome during the first 12 months. They were more frequently genotype‐positive (59%) than the whole group (32%) (Table S2). In real life, these patients with early events or with severe adverse events later may not reach the cardiogenetic care. They may be missed in studies recruiting patients retrospectively or mainly from out‐patient clinics. In contrast to previous reports, *TTNtv* were not associated with life‐threatening ventricular arrhythmias^37^ in the recent‐onset phase of the disease, reported in our study.

### Practical implications

Our data suggest that pathogenic and likely pathogenic non‐*TTN* variants, in addition to conventional clinical variables, can predict progression of heart failure after the initial evaluation of RODCM cases. Many events happened in the first year of follow‐up. This underscores the need for genetic testing with a rapid turnaround time. Carriers of non‐*TTN* variants should be then closely monitored.

Nonetheless, predicting life‐threatening ventricular arrhythmias in RODCM is notoriously difficult. Recent studies, including the DANISH trial,^38^ have questioned the benefits of primary preventive ICD implantation based solely on assessing LV ejection fraction in DCM patients on optimal medical therapy. New tools emerging in this field include assessment of late gadolinium enhancement and genetic testing, where there is strong evidence of arrhythmogenicity in lamin A/C, filamin C and *RBM20* variants even in subjects with mildly reduced LV ejection fraction.^39^ Our study revealed an increased risk of life‐threatening ventricular arrhythmias in carriers of class 4–5 non‐*TTN* VOIs. In agreement with previous studies, we observed that class 4–5 VOIs in genes coding nuclear envelope (mainly *LMNA*) and cytoskeletal proteins (mainly *DES and FLNC*) were associated with an increased risk of life‐threatening ventricular arrhythmias, as predicted from baseline assessment. In carriers of class 4–5 VOIs in genes coding nuclear envelope, an increased arrhythmic risk persisted even independent of 12‐months LV ejection fraction and presence of LVRR.

Taken together, the clinical contribution of genetic testing is most important in the first months after the diagnosis of RODCM, as there is the highest uncertainty regarding the disease outcomes. Importantly, prognostic consequences of genotyping of DCM patients have been highlighted in the latest guidelines of the European Society of Cardiology for the prevention of sudden cardiac death^39^ and management of cardiomyopathies.^40^

### Study limitations

There are several limitations to our study. First, the extreme genetic heterogeneity of RODCM complicated the correlation between VOIs of the selected DCM‐related genes, LVRR and clinical outcomes. Therefore, we had to divide patients into larger groups based on functional gene groups or use a crude division to *TTNtv* and *non‐TTN* VOIs. Second, genetic variables, except of the carrier status of class 4–5 VOIs of genes coding nuclear envelope, lost in our study a lot of their predictive strength after 12 months of follow‐up when compared with 12‐month variables including recent LVEF. They should be used mainly for early risk stratification after the baseline assessment. However, the initial months after the diagnosis of RODCM seem to be the most important period for improved risk stratification of life‐threatening ventricular arrhythmias. Third, routine cardiac magnetic resonance imaging was not a standard of care for patients with DCM at the creation of the study conception. Its inclusion into the study protocol was not possible from logistic and financial reasons. Forth, endomyocardial biopsy was performed only in selected patients, based on clinical judgement, to exclude curable causes of acute myocarditis. Finally, heart failure pharmacotherapy in our study group did not include sacubitril‐valsartan and sodium‐glucose cotransporter‐2 inhibitors, which were introduced into the clinical practice after completion of this study. Their effects of LVRR and prognosis in different genotypes of RODCM thus could not be evaluated.

## Conclusion

The monogenic genetic background can be traced in a substantial proportion of RODCM cases. At baseline assessment, carriers of pathogenic/likely pathogenic non*‐TTN* VOIs with RODCM have both an increased risk of death and progressive heart failure and also a burden of life‐threatening ventricular arrhythmias in comparison with genotype‐negative individuals. Among functional gene groups, pathogenic/likely pathogenic VOIs in genes coding nuclear envelope are strongly associated with both outcomes at baseline and also with life‐threatening ventricular arrhythmias after 12 months of follow‐up. In addition, VOIs in genes coding cytoskeleton are independently related to occurrence of life‐threatening ventricular arrhythmias at baseline assessment. Genetic testing can therefore improve early risk stratification after the initial evaluation of RODCM cases and contribute to individualized treatment of these patients.

## Conflict of interest

None declared.

## Funding

This study was supported by the Ministry of Health of the Czech Republic, the research grant (NV19‐08‐00122), MH CZ ‐ DRO (“Institute for Clinical and Experimental Medicine – IKEM, IN 00023001”), MH CZ ‐ DRO (FNOl, 00098892) and by the project National Institute for Research of Metabolic and Cardiovascular Diseases (Program EXCELES, Project No. LX22NPO5104) ‐ Funded by the European Union ‐ Next Generation EU. An additional funding was provided Charles University Institutional Programmes UNCE/MED/007, PROGRES‐Q26/LF1, and SVV2016/260148. All rights reserved. The authors would like to thank the Czech National Center for Medical Genomics (LM2018132) for their instrumental and technical support with the WES analyses.

## Supporting information


**Table S1.** On‐line databases used for selecting dilated cardiomyopathy (DCM) related genes by searching key word – ‘Dilated cardiomyopathy’. Table includes those 72 genes present at least in two databases. A pathogenic variant of PTPN11 was included based on the previous publication **(16)**.
**Table S2.** The first column shows results of genetic testing in the study group. The second and third columns compare genetic results of individuals who finished 12‐month follow‐up and those who had heart transplantation, implantation of left ventricular assist device or died before 12 months. In the third and fourth column are compared genetic results of individuals with and without left ventricular reverse remodelling at 12 months.
**Table S3.** Prediction of the LVRR at the final follow‐up (≥2 years) from the baseline and 12‐month data in a subgroup of 311 subjects with available long‐term echocardiographic follow‐up.
**Table S4.** Prediction of the primary and secondary outcome from baseline using univariate and multivariate Cox regression models. The primary endpoint represented the first event of all‐cause death, heart transplantation or implantation of ventricular assist device (VAD). The secondary outcome included the first event of sudden cardiac death, resuscitated cardiac arrest or treated ventricular tachyarrhythmia.
**Table S5.** Prediction of the primary and secondary outcome from 12 months of follow‐up using univariate and multivariate Cox regression models. The primary endpoint consisted of all‐cause death, heart transplantation or implantation of ventricular assist device (VAD) (52 events after 12 months). The secondary outcome included sudden cardiac death, resuscitated cardiac arrest or treated ventricular tachyarrhythmia (47 events after 12 months, including recurrence in 6 subjects after 12 months).

**Data S1.** Supporting Information.
