# Supplementary material for: Genotype is associated with left ventricular reverse remodelling and early events in recent‐onset dilated cardiomyopathy
Source: ESC Heart Fail. 2024 Aug 11;11(6):4127–38. doi: 10.1002/ehf2.15009 (PMC11631235; doi:10.1002/ehf2.15009)
Supplement: Supplementary file 1 — Table S1. On‐line databases used for selecting dilated cardiomyopathy (DCM) related genes by searching key word – ‘Dilated cardiomyopathy’. Table includes those 72 genes present at least in two databases. A pathogenic variant of PTPN11 was included based on the previous publication (16). Table S2. The first column shows results of genetic testing in the study group. The second and third columns compare genetic results of individuals who finished 12‐month follow‐up and those who had heart transplantation, implantation of left ventricular assist device or died before 12 months. In the third and fourth column are compared genetic results of individuals with and without left ventricular reverse remodelling at 12 months. Table S3. Prediction of the LVRR at the final follow‐up (≥2 years) from the baseline and 12‐month data in a subgroup of 311 subjects with available long‐term echocardiographic follow‐up. Table S4. Prediction of the primary and secondary outcome from baseline using univariate and multivariate Cox regression models. The primary endpoint represented the first event of all‐cause death, heart transplantation or implantation of ventricular assist device (VAD). The secondary outcome included the first event of sudden cardiac death, resuscitated cardiac arrest or treated ventricular tachyarrhythmia. Table S5. Prediction of the primary and secondary outcome from 12 months of follow‐up using univariate and multivariate Cox regression models. The primary endpoint consisted of all‐cause death, heart transplantation or implantation of ventricular assist device (VAD) (52 events after 12 months). The secondary outcome included sudden cardiac death, resuscitated cardiac arrest or treated ventricular tachyarrhythmia (47 events after 12 months, including recurrence in 6 subjects after 12 months). [file EHF2-11-4127-s001.docx]

**SUPPORTING INFORMATION 1:**

**LIST OF SUPPLEMENTAL MATERIALS (with page numbers):**

| **Supplemental Methods** |  | 1 |
| --- | --- | --- |
| **Supplemental Results** |  | 4 |
| **Supplemental Tables** |  | 5 |
|  | Supplemental Table 1 | 6 |
|  | Supplemental Table 2 | 8 |
|  | Supplemental Table 3 | 9 |
|  | Supplemental Table 4 | 12 |
|  | Supplemental Table 5 | 22 |
| **Supplemental References** |  | 30 |

**SUPPLEMENTAL METHODS:**

**Additional definitions**

Recent-onset dilated cardiomyopathy (RODCM) was defined as left ventricular (LV) or biventricular systolic dysfunction (defined as a left ventricular ejection fraction ˂45%) with or without LV dilatation unexplained by abnormal loading conditions or coronary artery disease **(1)**. Gender-specific thresholds for LV dilatation were based on an indexed LV end-diastolic diameter (>33 mm/m2 in males and >32 mm/m2 in females). Absence of LV dilatation defined the subgroup of patients with non-dilated hypokinetic cardiomyopathy **(1)**. Toxic cardiomyopathy was defined by a history of any chemotherapy and a history of prolonged and heavy alcohol consumption: that was, a self-reported history of alcohol intake of >80 g/day over a period of at least 5 years 2, 3, 6, with excess intake continuing up to no <3 months before initial diagnosis of RODCM **(2)**. Arrhythmia-induced cardiomyopathy was suspected in patients with mean heart rate >100 beats/min due to persistent atrial tachyarrhythmia and/or premature ventricular contractions burden ≥10% at disease presentation. The suspicion was confirmed in case of fast reversal of cardiomyopathy within 3 months after termination of tachyarrhythmia **(3).** Sudden cardiac death was defined as (i) witnessed death within one hour of the onset of any cardiac-related symptoms or (ii) nocturnal death with no antecedent history of immediate worsening of symptoms **(4)**. Equivalent doses of angiotensin-converting enzyme inhibitors, angiotensin receptor blockers and beta-blockers were expressed as a percentage of the maximum recommended daily dose according to the guidelines **(5)**.

**Technical details of genetic analysis**

Genomic DNA was extracted from peripheral blood leukocytes using a standard technology. WES was performed on 1 µg of DNA in all individuals tested. For DNA enrichment, barcoded DNA libraries and SeqCap EZ MedExome Target Enrichment Kit (SeqCap EZ MedExome Probes, Roche, Madison, USA) were used according to the manufacturer’s protocol. MPS was performed on the captured barcoded DNA library using the Illumina HiSeq 2500 system at the genomic facility at the National Coordination Centre for Rare Diseases (www.nkcvo.cz) at Motol University Hospital; Prague. The resulting FASTQ files were aligned to the Human Genome Reference (hg19) using NovoAlign software (ver. 2.08.03; www.novocraft.com). Following genome alignment, conversion of SAM format to BAM and duplicate removal were performed using Picard Tools (ver. 1.129; broadinstitute.github.io/picard/). The Genome Analysis Toolkit, GATK (3.7) **(6)** was used for local realignment around indels, base recalibration and variant recalibration and genotyping. Variant annotation was performed with SnpEff **(7)** and GEMINI **(8)**. Rare variants were defined as having a frequency ≤ 0.05% among control samples. Comparisons were made with ethnically matched controls available from the database of genomics variants maintained by the Czech National Center for Medical Genomics; (http://ncmg.cz/en) and the publicly available genotype data from subjects of European origin sequenced in the 1000 Genomes Project, Exome Aggregation Consortium (ExAC) and The Genome Aggregation Database (gnomAD) **(9)**. Identified genetic variants were filtered according to the expected autosomal dominant model of disease inheritance and evaluated according to the biological relevance of corresponding candidate genes. Candidate variants were visualized in Integrative Genomics Viewer (IGV; ver. 2.3.32) **(10,11)**.

**Statistical analysis**

Categorical data were expressed as percentages and compared using chi-square analysis or Fisher exact tests. Normally distributed continuous variables were expressed as a mean and standard deviation. Abnormally distributed continuous variables were given as a median and interquartile range. They were compared using the Student t-test for paired or unpaired data, or by the non-parametric Mann-Whitney test where appropriate. Univariate Cox regression models were used to identify predictors of the primary and secondary outcomes among 81 baseline and 36 12-month variables. Variables that were significant on univariate analysis (p<0.1) were entered into multivariable Cox regression models using forwards and backwards stepwise elimination. Only those variables found to be consistent between each method were included in the final model. To reduce the risk of overfitting the model to the dataset, we used a stepwise approach and included in multivariate models less than one variable per ten events **(12)**. For all tests, a probability value of p<0.05 was considered significant. The analysis was performed using statistical software SPSS (Chicago, Illinois, USA) for Windows, version 17.0.

**SUPPLEMENTAL RESULTS**

**Spectrum of implanted devices and the mode of death**

In addition to optimizing heart failure pharmacotherapy, a single or dual-chamber ICD, a biventricular ICD and a biventricular pacemaker was implanted in 99 (26%), 42 (11%) and 6 (1.5%) subjects, respectively. The median time from baseline to implantation was 11 months (1-23). The mode of death was progression of heart failure in 13 patients, sudden cardiac death in 10 patients (including 4 individuals with ICD), non-cardiac death in 4 patients (suicide, brain hemorrhage, pancreatic tumor, sepsis) and unknown in 5 patients.

**Previously published variants**

We published detailed genotype-phenotype correlates of the following variants previously (*FKTN –* deletion of exons 1-9 together with a missense variant *FKTN* p.His172Leu*; DES* p. Ser57Leu; *LAMP2*- deletion of *CUL4B, LAMP2, ATP1B4, TMEM255A*, and *ZBTB33*) **(13-15)**.

**SUPPLEMENTAL TABLES**

**Supplemental Table 1:** On-line databases used for selecting dilated cardiomyopathy (DCM) related genes by searching key word – “Dilated cardiomyopathy”. Table includes those 72 genes present at least in two databases. A pathogenic variant of PTPN11 was included based on the previous publication **(16)**.

1. OMIM – <https://omim.org/>
2. ClinVar – <https://www.ncbi.nlm.nih.gov/clinvar/>
3. HPO – <https://hpo.jax.org/app/download/annotation>
4. Orphanet – <http://www.orphadata.org/cgi-bin/index.php>
5. HGMD – <https://portal.biobase-international.com/hgmd/pro/search_gene.php>
6. GeneCards – <https://www.genecards.org/>;score >40

| Fuctional annotation | Gene | Gene name |
| --- | --- | --- |
| Sarcomere | *ACTC1*  *MYBPC3*  *MYH6*  *MYH7*  *MYL2*  *MYL3*  *MYLK2*  *MYOM1*  *MYOZ2*  *TNNC1*  *TNNI3*  *TNNI3K*  *TNNT2*  *TPM1*  *TTN* | α-Cardiac actin  Myosin-binding protein C  α-Myosin heavy chain  β-Myosin heavy chain  Myosin light chain 2, regulatory  Myosin light chain 3  Myosin light chain kinase 2  Myomesin 1  Myozenin 2  Cardiac troponin C  Cardiac troponin I  Troponin I–interacting kinase  Cardiac troponin T  α-Tropomyosin  Titin |
| Z-disk | *ACTN2*  *ANKRD1*  *BAG3*  *CRYAB*  *CSRP3*  *FHL2*  *LDB3*  *MYPN*  *NEXN*  *NEBL*  *TCAP* | α-Actinin 2  Cardiac ankyrin repeat protein  BCL2-associated athanogene 3  α-B-crystallin  Muscle LIM protein  Four-and-a-half LIM protein 2  Cypher/ZASP  Myopalladin  Nexilin  Nebulette  Titin-cap/telethonin |
| Dystrophin complex | *DMD*  *DTNA*  *FKRP*  *FKTN*  *SGCB*  *SGCD* | Dystrophin  α-Dystrobrevin  Fukutin-related protein  Fukutin  β-Sarcoglycan  δ-Sarcoglycan |
| Cytoskeleton | *ACTA1*  *DES*  *FLNC*  *VCL* | α-Skeletal actin  Desmin  Filamin C  Vinculin |
| Desmosomes | *DSC2*  *DSG2*  *DSP*  *JUP*  *PKP2* | Desmocollin 2  Desmoglein 2  Desmoplakin  Junction plakoglobin  Plakophilin 2 |
| Sarcoplasmic reticulum and Cytoplasm | *DOLK*  *JPH2*  *PLN*  *PRKAG2*  *RAF1*  *RYR2* | Dolichol kinase  Junctophilin 2  Phospholamban  Protein kinase AMP-activated noncatalytic subunit 2 γ  Proto-oncogene  Ryanodine receptor 2, Ca channel |
| Nuclear envelope | *EMD*  *LMNA*  *SYNE1*  *SYNE2*  *TMPO* | Emerin  Lamin A/C  Nesprin 1  Nesprin 2  Thymopoietin |
| Nucleus | *EYA4*  *GATAD1*  *NKX2-5*  *PLEKHM2*  *PRDM16*  *RBM20*  *TBX20*  *TGFB3*  *TMEM43* | EYA transcriptional coactivator and phosphatase 4  GATA zinc finger domain containing 1  Cardiac-specific homeobox 1  Pleckstrin homology domain  PR/SET domain 16  RNA Binding motif Protein 20  T-box transcription factor 20  Transforming growth factor β 3  Transmembrane Protein 43 |
| Ion channels | *ABCC9*  *SCN5A* | ATP Binding cassette subfamily C member 9  Type V voltage-gated cardiac Na channel |
| Mitochondria | *DNAJC19*  *mtDNA*  *SDHA*  *TXNRD2*  *TAZ* | HSP40 homolog, C19  Mitochondrial-encoded TRNA genes  Succinate dehydrogenase  Thioredoxin reductase 2  Tafazzin |
| Extracellular matrix | *LAMA4* | Laminin 4 |
| Lysosome | *LAMP2* | Lysosome-associated membrane protein 2 |
| Other | *PSEN1*  *PSEN2* | Presenillin 1  Presenillin |

**Supplemental Table 2:** The first column shows results of genetic testing in the study group. The second and third columns compare genetic results of individuals who finished 12-month follow-up and those who had heart transplantation, implantation of left ventricular assist device or died before 12 months. In the third and fourth column are compared genetic results of individuals with and without left ventricular reverse remodeling at 12 months.

|  | Baseline characteristics  (n=386) | Baseline data, Finished 12-month follow-up  (n=359) | Baseline data,  HTx, VAD or death before 12 months  (n=27) | Baseline data,  LVRR present at 12 months  (n=171) | Baseline data,  LVRR absent at 12 months  (n=188) |
| --- | --- | --- | --- | --- | --- |
| Familial DCM | 98 (25%) | 90 (25%) | 8 (30%) | 26 (15%) | 64 (34%) *** |
| Negative result of WES | 158 (41%) | 151 (42%) | 7 (26%) | 80 (47%) | 71 (38%) |
| Variants of unknown significance only | 103 (27%) | 99 (28%) | 4 (15%) | 47 (27%) | 52 (28%) |
| Single *TTNtv* class 4-5 | 69 (18%) | 63 (18%) | 6 (22%) | 31 (18%) | 32 (17%) |
| Non-titin VOIs class 4-5 | 56 (14%) | 46 (12%) | 10 (37%) ** | 13 (8%) | 33 (18%) ** |
| VOIs class 4-5 in genes coding nuclear envelope | 8 (3%) | 5 (1.4%) | 3 (11%)* | 3 (1.6%) | 2 (1.2%) |
| VOIs class 4-5 in genes coding nuclear components | 8 (2.1%) | 8 (2.2%) | 0 (0%) | 7 (3.6%) | 1 (0.6%) |

P-value was coded: p<0.05 *, p<0.01 **, p<0.001 ***.

**Abbreviations:** DCM= dilated cardiomyopathy, HTx= heart transplantation, *TTNtv*= titin truncating variant, VAD= ventricular assist device, VOIs= variants of interest, WES = whole-exome sequencing

**Supplemental Table 3:** Prediction of the LVRR at the final follow-up (≥2 years) from the baseline and 12-month data in a subgroup of 311 subjects with available long-term echocardiographic follow-up.

|  | Baseline data,  LVRR present at the last FU  (n=173) | Baseline data,  LVRR absent at the last FU  (n=138) | 12-month data,  LVRR present at the last FU  (n=173) | 12-month data,  LVRR absent at the last FU  (n=138) |
| --- | --- | --- | --- | --- |
| Age (years) | 45±13 | 46±12 | - | - |
| Males | 121 (70%) | 104 (75%) | - | - |
| Diabetes mellitus | 16 (9%) | 17 (12%) | - | - |
| Arterial hypertension | 49 (28%) | 38 (27%) | - | - |
| Asthma bronchiale | 10 (6%) | 10 (7%) | - | - |
| History of persistent atrial fibrillation | 19 (11%) | 28 (20%)* | - | - |
| Viral prodroms | 67 (39%) | 54 (39%) | - | - |
| Decompensated HF at  admission | 63 (36%) | 57 (41%) | - | - |
| Manifestation by sustained ventricular tachyarrhythmia | 2 (1%) | 5 (2%) |  |  |
| NYHA class I  II  III  IV | 11 (7%)  103 (59%)  45 (26%)  14 (8%) | 10 (7%)  76 (55%)  45 (33%)  7 (5%) | 82 (47%)  84 (49%)  7 (4%)  0 | 47 (34%) **  72 (52%)  19 (14 %)  0 |
| ACEI or ARB | 145 (84%) | 111 (80%) | 147 (86%) | 122 (88%) |
| ACEI/ARB ≥ 50% of recommended dose (%) | 70 (40%) | 57 (41%) | 95 (55%) | 72 (52%) |
| Beta-blockers | 151 (88%) | 111 (86%) | 169 (99%) | 137 (99%) |
| Beta-blockers ≥ 50% of recommended dose (%) | 56 (32%) | 38 (28%) | 124 (72%) | 86 (62%) |
| Aldosteron receptor  blockers | 125 (73%) | 91 (66%) | 129 (75%) | 100 (72%) |
| Furosemide | 143 (83%) | 104 (75%) | 132 (76%) | 108 (78%) |
| Furosemide ≥ 40 mg/day (%) | 109 (63%) | 74 (54%) | 75 (43%) | 63 (46%) |
| Digoxin | 7 (4%) | 19 (14%) ** | 11 (6%) | 17 (12%) |
| Intravenous diuretics | 29 (17%) | 18 (13%) | 0 | 0 |
| Inotropes | 7 (2%) | 11 (4%) | 0 | 0 |
| BMI (kg/m^2^) | 29±12 | 27±6 | 29±5 | 28±6 |
| Systolic BP (mm Hg) | 120±19 | 120±17 | 125±20 | 119±18 ** |
| Diastolic BP (mm Hg) | 77±13 | 76±11 | 79±13 | 77±12 |
| Heart rate (bpm) | 85±17 | 81±16 | 69±12 | 71±14 |
| Sinus rhythm | 170 (98%) | 134 (98%) | 167 (98%) | 126 (91%) |
| QRS duration (ms) | 102±23 | 111±29 ** | 106±25 | 115±30 ** |
| Complete LBBB | 29 (17%) | 30 (22%) | 22 (13%) | 25 (18%) |
| LVEDD (mm) | 66±7 | 68±8 | 59±7 | 65±8 *** |
| LVEDD (mm/m^2^) | 32±5 | 34±5 * | 29±4 | 33±5 *** |
| Interventricular septum (mm) | 9±2 | 9±2 | 10±2 | 9±2 |
| Posterior wall (mm) | 9±1 | 9±2 | 9±2 | 9±1 |
| LVEF (%) | 24±7 | 26±8 ** | 42±10 | 31±10 *** |
| LVEF˃ 35% | 11 (6%) | 17 (12%) | 129 (75%) | 46 (33%) *** |
| LVEF˃ 50% | - | - | 33 (19%) | 6 (4%) *** |
| LVRR at 12M | - | - | 122 (71%) | 26 (19%) *** |
| Restrictive mitral pattern (n=244) | 50 (21%) | 41 (19%) | 4 (3%) | 17 (16%) *** |
| E/E´ ratio  (BL n=265, FU n=277) | 13.5±6.5 | 12.9±6.1 | 8.0±2.7 | 10.6±4.6 *** |
| Left atrium short axis (mm) | 46±7 | 46±7 | 40±6 | 43±7 ** |
| Left atrium long axis (mm)  (BL n=210, FU n=216) | 57±11 | 58±9 | 51±8 | 54±8 ** |
| LAVI (ml/m^2^)  (BL n=192, FU n= 87) | 46±16 | 49±19 | 32±11 | 41±14 *** |
| Mitral regurgitation ≥  moderate | 45 (26%) | 41 (30%) | 8 (5%) | 23 (17%) *** |
| RVD1 (mm)  (BL n=222, FU n=161) | 36±6 | 37±6 | 35±5 | 36±5 |
| Tricuspid annulus Sm  (BL n=265, FU n=259) | 9.8±2.8 | 10.4±3.3 | 12.0±2.5 | 11.3±2.3 |
| TAPSE (mm)  (BL n=262, FU n=271) | 18±4 | 19±4 | 22±4 | 20±4 * |
| Tricuspid regurgitation ≥ moderate | 15 (9%) | 17 (12%) | 0 | 4 (3%) |
| Sodium (mmol/L)  (BL n= 301 , FU n= 221) | 139.6±2.9 | 139.4±3.4 | 138.8±3.0 | 138.9±3.0 |
| Creatinine (μmol/L)  (BL n= 311, FU n= 226) | 92±24 | 88±18 | 86±23 | 88±17 |
| Estimated GFR (ml/min)  (BL n= 311) | 111±38 | 111±39 | - | - |
| B-type natriuretic peptide (ng/L)  (BL n=201, FU n=101) | 288 (105-709) | 267 (82-562) | 30 (14-60) | 118 (33-302) *** |
| NT-proBNP (ng/L)  (BL n=77, FU n=67) | 1678 (741-2527) | 1527 (733- 3737) | 161 (79-537) | 643 (199-1130) *** |
| BNP/NT-proBNP quartile:  1st  2nd  3rd  4th  (BL n=278) | 43 (27%)  36 (23%)  46 (30%)  31 (20%) | 32 (26%)  35 (29%)  26 (21%)  29 (24%) | - | - |
| Hs-cTNT (ng/L)  (BL n=160) | 13.0 (8.0-22.7) | 17.2 (10.3-30) | - | - |
| Troponin I (μg/l)  (BL n=77) | 0.03 (0.00-0.06) | 0.025 (0.00-0.07) | - | - |
| Troponin I > 0.03 or hs-cTNT >13.5 ng/L  (BL n=209) | 58 (46%) | 39 (47%) | - | - |
| Familial DCM | 31 (18%) | 44 (32%) ** | - | - |
| Negative result of WES | 77 (45%) | 58 (42%) | - | - |
| Variants of unknown significance only | 52 (30%) | 37 (27%) | - | - |
| Single *TTNtv* VOIs class 4-5 | 36 (21%) | 21 (15%) | - | - |
| Non-titin VOIs class 4-5 | 8 (5%) | 22 (16%) ** | - | - |
| VOIs class 4-5 in genes coding nuclear envelope | 1 (0.6%) | 2 (1.4%) | - | - |
| VOIs class 4-5 in genes coding nuclear components | 0 (0%) | 7 (5%) ** | - | - |

P-value was coded: p<0.05 *, p<0.01 **, p<0.001 ***.

**Abbreviations:** ACEI= angiotensin converting enzyme inhibitors, ARB= angiotensin receptor blockers, BMI= body mass index, BNP= B-type natriuretic peptide, BP= blood pressure, DCM= dilated cardiomyopathy, GFR= glomerular filtration rate, Hs-cTNT= high sensitivity cardiac troponin T, LBBB= left bundle branch block, LVEDD= left ventricular end-diastolic dimension, LVEF= left ventricular ejection fraction, LAVI= left atrial volume index, NT-proBNP= N-terminal pro-B-type natriuretic peptide, RVD1= basal right ventricular diameter, TAPSE= tricuspid annular systolic plane excursion, *TTNtv* = titin truncating variant, WES = whole-exome sequencing.

**Supplemental Table 4:** Prediction of the primary and secondary outcome from baseline using univariate and multivariate Cox regression models. The primary endpoint represented the first event of all-cause death, heart transplantation or implantation of ventricular assist device (VAD). The secondary outcome included the first event of sudden cardiac death, resuscitated cardiac arrest or treated ventricular tachyarrhythmia.

| **1. Baseline univariate predictors of the primary outcome (all cause mortality,**  **heart transplantation or implantation of VAD)** | | | | | | |
| --- | --- | --- | --- | --- | --- | --- |
| **Variable** | **Chi-square** | **p-value** | **Category for calculation of hazard ratios** | **Hazard ratio** | **95% CI** | **Pairwise comparison**  **(p-value)** |
| Age (years) | 16.53 | 0.000*** | Per unit increase | 0.96 | 0.95-0.98 |  |
| Sex | 4.85 | 0.028* | Males vs. females | 1.88 | 1.06-3.33 |  |
| History of arterial hypertension | 6.12 | 0.013* | Present vs. absent | 0.44 | 0.23-0.86 |  |
| History of persistent atrial fibrillation | 5.95 | 0.015* | Present vs. absent | 1.82 | 1.12-2.96 |  |
| Decompensated heart failure at baseline | 17.13 | 0.000*** | Present vs. absent | 2.59 | 1.63-4.13 |  |
| NYHA class | 32.97 | 0.000*** | Class II vs. I  Class III vs. I  Class IV vs. I | 1.78  4.42  10.29 | 0.418-7.55  1.06-18.36  2.34-45.30 | 0.437  0.041*  0.002** |
| Use of inotropes | 71.10 | 0.000*** | Present vs. absent | 6.25 | 3.86-10.25 |  |
| Heart rate (bpm) | 7.06 | 0.007** | Per unit increase | 1.02 | 1.01-1.03 |  |
| LVEDD (mm/m^2^) | 13.88 | 0.000*** | Per unit increase | 1.08 | 1.04-1.12 |  |
| LVEF (%) | 16.43 | 0.000*** | Per unit increase | 0.93 | 0.90-0.96 |  |
| TAPSE (mm) (n=321) | 14.57 | 0.000*** | Per unit increase | 0.88 | 0.82-0.94 |  |
| LAVI (ml/m^2^) (n=241) | 30.57 | 0.000*** | Per unit increase | 1.04 | 1.02-1.05 |  |
| Mitral regurgitation ≥ moderate | 16.94 | 0.000*** | Present vs. absent | 2.49 | 1.59-3.90 |  |
| Quartile of BNP/NT-proBNP  (n=345) | 43.99 | 0.000*** | 2^nd^ vs 1^st^ quartile  3^rd^ vs 1^st^ quartile  4^th^ vs 1^st^ quartile | 1.33  3.28  9.11 | 0.42-4.18  1.20-8.95  3.56-23.31 | 0.630  0.021*  0.000*** |
| Main categories of VOIs (negative, VUS, *TTNtv* class 4-5, non-titin class 4-5) | 11.02 | 0.012* | VUS vs. negative  Titin vs. negative  Non-titin vs. negative | 0.96  1.28  2.36 | 0.51-1.82  0.68-2.42  1.32-4.20 | 0.910  0.451  0.004** |
| Negative result of WES | 2.05 | 0.125 | Negative vs. others | 0.71 | 0.44-1.14 |  |
| VOIs class 4-5 in non-titin genes vs. others | 10.39 | 0.001** | Non-titin vs. others | 2.24 | 1.36-3.72 |  |
| VOIs class 4-5 in genes coding nuclear envelope | 17.36 | 0.003 *** | Nuclear envelope vs. others | 5.60 | 2.24-13.96 |  |
| **2. Baseline univariate predictors of the secondary outcome (sudden cardiac death, resuscitated cardiac arrest or treated ventricular tachyarrhythmia).** | | | | | | |
| **Variable** | **Chi-square** | **p-value** | **Category for calculation of hazard ratios** | **Hazard ratio** | **95% CI** | **Pairwise comparison**  **(p-value)** |
| Decompensated heart failure at baseline | 4.76 | 0.029* | Present vs. absent | 1.90 | 1.06-4.13 |  |
| Ventricular tachyarrhythmia at presentation | 4.56 | 0.033* | Present vs. absent | 2.91 | 1.04-3.42 |  |
| LVEDD (mm/m^2^) | 6.39 | 0.011* | Per 10 units increase | 1.98 | 1.17-3.37 |  |
| LVEF (%) | 4.12 | 0.042* | Per unit increase | 0.96 | 0.92-0.99 |  |
| RVD1 (mm) (n=281) | 4.16 | 0.041* | Per unit increase | 1.05 | 1.002-1.11 |  |
| TAPSE (mm) (n=321) | 5.48 | 0.019* | Per unit increase | 0.92 | 0.85-0.99 |  |
| LAVI (ml/m^2^) (n=241) | 11.82 | 0.001** | Per unit increase | 1.03 | 1.16-1.05 |  |
| Mitral regurgitation ≥ moderate | 4.91 | 0.027* | Present vs. absent | 1.88 | 1.07-3.32 |  |
| Quartile of BNP/NT-proBNP (n=345) | 8.23 | 0.041* | 2^nd^ vs 1^st^ quartile  3^rd^ vs 1^st^ quartile  4^th^ vs 1^st^ quartile | 1.02  1.20  2.53 | 0.41-2.51  0.47-2.95  1.11-5.27 | 0.969  0.696  0.026 * |
| Main categories of VOIs (negative, VUS, *TTNtv* class 4-5, non-titin class 4-5) | 9.61 | 0.022* | VUS vs. negative  *TTNtv* vs. negative  Non-titin vs. negative | 1.85  1.26  3.08 | 0.89-3.85  0.53-3.01  1.42-6.67 | 0.077  0.599  0.004** |
| Negative result of WES | 4.73 | 0.030* | Negative vs. others | 0.51 | 0.28-0.95 |  |
| VOIs class 4-5 in non-titin genes | 7.16 | 0.007** | Non-titin vs. others | 2.37 | 1.23-4.55 |  |
| VOIs class 4-5 in genes coding nuclear envelope | 16.89 | 0.000*** | Positive vs. negative | 7.99 | 2.46-25.97 |  |
| VOIs class 4-5 in genes coding cytoskeleton | 10.33 | 0.001** | Positive vs. negative | 5.53 | 1.71-17.90 |  |
| **3. Baseline multivariate models predicting the primary outcome (all cause mortality, heart transplantation or implantation of VAD)**  Variables in multivariate models:  A) Age, sex, history of arterial hypertension, LVEDD (mm/m2), LVEF (%), quartile of BNP/NT-proBNP, main categories of VOIs (negative, VUS, *TTNtv* class 4-5, non-titin class 4-5)  B) Age, sex, history of arterial hypertension, LVEDD (mm/m2), LVEF (%), quartile of BNP/NT-proBNP, VOIs class 4-5 in non-titin genes vs. others  C) Age, sex, history of arterial hypertension, LVEDD (mm/m2), LVEF (%), use of inotropes, main categories of VOIs (negative, VUS, *TTNtv* class 4-5, non-titin class 4-5)  D) Age, sex, history of arterial hypertension, LVEDD (mm/m2), LVEF (%), use of inotropes, VOIs class 4-5 in non-titin genes  E) Age, sex, history of arterial hypertension, LVEDD (mm/m2), LVEF (%), quartile of BNP/NT-proBNP, VOIs class 4-5 in genes coding nuclear envelope  F) Age, sex, history of arterial hypertension, LVEDD (mm/m2), LVEF (%), use of inotropes, VOIs class 4-5 in genes coding nuclear envelope | | | | | | |
|  | **Wald score** | **p-value** | **Category for calculation of hazard ratios** | **Hazard ratio** | **95% CI** | **Pairwise comparison**  **(p-value)** |
| **Model A)**  Age (years) | 8.63 | 0.003** | Per unit increase | 0.97 | 0.95-0.99 |  |
| Sex | 4.015 | 0.045* | Males vs. females | 1.99 | 1.02-3.90 |  |
| Quartile of BNP/NT-proBNP  (n=345) | 39.50 | 0.000*** | 2^nd^ vs 1^st^ quartile  3^rd^ vs 1^st^ quartile  4^th^ vs 1^st^ quartile | 1.72  3.43  10.68 | 0.54-5.50  1.25-9.39  4.11-27.71 | 0.361  0.016*  0.000 *** |
| Main categories of VOIs (negative, VUS, *TTNtv* class 4-5, non-titin class 4-5) | 12.97 | 0.005** | VUS vs. negative  Titin vs. negative  Non-titin vs. negative | 0.89  0.82  2.85 | 0.44-1.80  0.40-1.64  1.44-5.66 | 0.751  0.567  0.003** |
| **Model B)**  Age (years) | 8.55 | 0.003** | Per unit increase | 0.97 | 0.95-0.99 |  |
| Sex | 3.97 | 0.046* | Males vs. females | 1.97 | 1.01-3.86 |  |
| Quartile of BNP/NT-proBNP  (n=345) | 39.82 | 0.000*** | 2^nd^ vs 1^st^ quartile  3^rd^ vs 1^st^ quartile  4^th^ vs 1^st^ quartile | 1.72  3.40  10.60 | 0.54-5.49  1.24-9.31  4.09-27.46 | 0.361  0.016*  0.000 *** |
| VOIs class 4-5 in non-titin genes | 12.70 | 0.000*** | Non-titin vs. others | 3.10 | 1.66-5.77 |  |
| **Model C)**  Age (years) | 7.38 | 0.007** | Per unit increase | 0.97 | 0.96-0.99 |  |
| Sex | 6.43 | 0.011* | Males vs. females | 2.20 | 1.20-4.06 |  |
| LVEF (%) | 8.18 | 0.004** | Per unit increase | 0.95 | 0.92-0.98 |  |
| Use of inotropes | 29.91 | 0.000*** | Present vs. absent | 4.25 | 2.53-7.13 |  |
| Main categories of VOIs (negative, VUS, *TTNtv* class 4-5, non-titin class 4-5) | 12.54 | 0.006** | VUS vs. negative  Titin vs. negative  Non-titin vs. negative | 0.97  1.12  2.69 | 0.51-1.85  0.59-2.16  1.48-4.91 | 0.751  0.567  0.003** |
| **Model D)**  Age (years) | 7.75 | 0.005** | Per unit increase | 0.97 | 0.96-0.99 |  |
| Sex | 6.50 | 0.011* | Males vs. females | 2.21 | 1.20-4.05 |  |
| LVEF (%) | 8.61 | 0.003** | Per unit increase | 0.95 | 0.92-0.98 |  |
| Use of inotropes | 30.30 | 0.000*** | Present vs. absent | 4.21 | 2.52-7.01 |  |
| VOIs class 4-5 in non-titin genes | 12.41 | 0.000*** | Non-titin vs. others | 2.64 | 1.54-4.53 |  |
| **Model E)**  Age (years) | 11.88 | 0.001** | Per unit increase | 0.97 | 0.95-0.99 |  |
| Quartile of BNP/NT-proBNP  (n=345) | 37.44 | 0.000*** | 2^nd^ vs 1^st^ quartile  3^rd^ vs 1^st^ quartile  4^th^ vs 1^st^ quartile | 1.67  3.65  9.71 | 0.53-5.33  1.33-10.02  3.79-24.90 | 0.383  0.012*  0.000 *** |
| VOIs class 4-5 in genes coding nuclear envelope | 7.59 | 0.006** | Nuclear envelope vs. others | 5.29 | 1.62-17.30 |  |
| **Model F)**  Age (years) | 10.89 | 0.001** | Per unit increase | 0.97 | 0.95-0.99 |  |
| LVEF (%) | 7.55 | 0.006** | Per unit increase | 0.95 | 0.92-0.99 |  |
| Use of inotropes | 33.47 | 0.000*** | Present vs. absent | 4.59 | 2.74-7.69 |  |
| VOIs class 4-5 in genes coding nuclear envelope | 20.33 | 0.006** | Nuclear envelope vs. others | 8.55 | 3.36-21.73 |  |
| **4. Baseline multivariate models predicting the secondary outcome (sudden cardiac death, resuscitated cardiac arrest or treated ventricular tachyarrhythmia)**  Variables in multivariate models:  A) Manifestation by sustained ventricular arrhythmia, LVEDD (mm/m2), LVEF (%), quartile of BNP/NT-proBNP, main categories of VOIs (negative, VUS, *TTNtv* class 4-5, non-titin class 4-5)  B) Manifestation by sustained ventricular arrhythmia, LVEDD (mm/m2), LVEF (%), quartile of BNP/NT-proBNP, negative result of WES  C) Manifestation by sustained ventricular arrhythmia, LVEDD (mm/m2), LVEF (%), quartile of BNP/NT-proBNP, VOIs class 4-5 in non-titin genes  D) Manifestation by sustained ventricular arrhythmia, LVEDD (mm/m2), LVEF (%), quartile of BNP/NT-proBNP, VOIs class 4-5 in genes coding nuclear envelope  E) Manifestation by sustained ventricular arrhythmia, LVEDD (mm/m2), LVEF (%), quartile of BNP/NT-proBNP, VOIs class 4-5 in genes coding cytoskeleton  F) Manifestation by sustained ventricular arrhythmia, LVEDD (mm/m2), LVEF (%), main categories of VOIs (negative, VUS, *TTNtv* class 4-5, non-titin class 4-5)  G) Manifestation by sustained ventricular arrhythmia, LVEDD (mm/m2), LVEF (%), negative result of WES  H) Manifestation by sustained ventricular arrhythmia, LVEDD (mm/m2), LVEF (%), VOIs class 4-5 in non-titin genes  I) Manifestation by sustained ventricular arrhythmia, LVEDD (mm/m2), LVEF (%), VOIs class 4-5 in genes coding nuclear envelope  J) Manifestation by sustained ventricular arrhythmia, LVEDD (mm/m2), LVEF (%), VOIs class 4-5 in genes coding cytoskeleton | | | | | | |
|  | **Wald score** | **p-value** | **Category for calculation of hazard ratios** | **Hazard ratio** | **95% CI** | **Pairwise comparison**  **(p-value)** |
| **Model A)**  Quartile of BNP/NT-proBNP  (n=345) | 11.36 | 0.010* | 2^nd^ vs 1^st^ quartile  3^rd^ vs 1^st^ quartile  4^th^ vs 1^st^ quartile | 1.13  1.19  3.31 | 0.46-2.81  0.48-2.93  1.42-7.70 | 0.784  0.709  0.005** |
| Main categories of VOIs (negative, VUS, *TTNtv* class 4-5, non-titin class 4-5) | 11.97 | 0.005** | VUS vs. negative  Titin vs. negative  Non-titin vs. negative | 1.93  1.10  4.37 | 0.90-4.12  0.44-2.77  1.90-10.05 | 0.090  0.834  0.001** |
| **Model B)**  Baseline LVEDD (mm/m2) | 5.66 | 0.017* | Per 10 unit increase | 1.96 | 1.13-3.42 |  |
| Negative result of WES | 4.89 | 0.027* | Negative vs. others | 0.48 | 0.25-0.92 |  |
| **Model C)**  Quartile of BNP/NT-proBNP  (n=345) | 10.74 | 0.013* | 2^nd^ vs 1^st^ quartile  3^rd^ vs 1^st^ quartile  4^th^ vs 1^st^ quartile | 1.12  1.20  2.63 | 0.45-2.77  0.49-2.95  1.16-5.96 | 0.805  0.714  0.007** |
| VOIs class 4-5 in non-titin genes | 11.22 | 0.001** | Non-titin vs. others | 3.38 | 1.66-6.89 |  |
| **Model D)**  Quartile of BNP/NT-proBNP  (n=345) | 8.86 | 0.031* | 2^nd^ vs 1^st^ quartile  3^rd^ vs 1^st^ quartile  4^th^ vs 1^st^ quartile | 1.15  1.32  2.91 | 0.46-2.88  0.53-2.39  1.26-6.74 | 0.759  0.547  0.012* |
| VOIs class 4-5 in genes coding nuclear envelope | 13.96 | 0.000*** | Nuclear envelope vs. others | 10.10 | 3.00-33.99 |  |
| **Model E)**  Quartile of BNP/NT-proBNP  (n=345) | 8.35 | 0.039* | 2^nd^ vs 1^st^ quartile  3^rd^ vs 1^st^ quartile  4^th^ vs 1^st^ quartile | 1.03  1.12  2.61 | 0.42-2.54  0.45-2.78  1.15-5.93 | 0.944  0.803  0.022* |
| VOIs class 4-5 in genes coding cytoskeleton | 8.54 | 0.003** | Present vs. absent | 5.18 | 1.60-16.81 |  |
| **Model F)**  Baseline LVEDD (mm/m2) | 6.70 | 0.010* | Per 10 unit increase | 2.02 | 1.19-3.43 |  |
| Main categories of VOIs (negative, VUS, *TTNtv* class 4-5, non-titin class 4-5) | 10.42 | 0.015* | VUS vs. negative  Titin vs. negative  Non-titin vs. negative | 1.99  1.38  3.33 | 0.96-4.15  0.58-3.32  1.56-7.08 | 0.064  0.466  0.002** |
| **Model G)**  Baseline LVEDD (mm/m2) | 7.83 | 0.005** | Per 10 unit increase | 2.13 | 1.25-3.60 |  |
| Negative result of WES | 5.53 | 0.019* | Negative vs. others | 0.48 | 0.26-0.88 |  |
| **Model H)**  Baseline LVEDD (mm/m2) | 5.81 | 0.016* | Per 10 unit increase | 1.94 | 1.13-3.32 |  |
| VOIs class 4-5 in non-titin genes | 7.75 | 0.005** | Non-titin vs. others | 2.46 | 1.31-4.63 |  |
| **Model I)**  Baseline LVEDD (mm/m2) | 6.16 | 0.013* | Per 10 unit increase | 1.97 | 1.15-3.36 |  |
| VOIs class 4-5 in genes coding nuclear envelope | 11.35 | 0.001** | Nuclear envelope vs. others | 7.61 | 2.34-24.80 |  |
| **Model J)**  Baseline LVEDD (mm/m2) | 6.12 | 0.013* | Per 10 unit increase | 1.97 | 1.15-3.38 |  |
| VOIs class 4-5 in genes coding cytoskeleton | 7.58 | 0.006** | Cytoskeleton vs. others | 5.21 | 1.61-16.85 |  |

**Abbrevations:** ACMG= American College of Medical Genetics and Genomics, BNP= B-type natriuretic peptide, LVEDD= left ventricular end-diastolic dimension, LVEF= left ventricular ejection fraction, LAVI= left atrial volume index, NT-proBNP= N-terminal pro-B-type natriuretic peptide, RVD1= basal right ventricular diameter, TAPSE= tricuspid annular systolic plane excursion, *TTNtv* = titin truncating variant, VOIs= variants of interest, WES = whole-exome sequencing

**Supplemental Table 5:** Prediction of the primary and secondary outcome from 12 months of follow-up using univariate and multivariate Cox regression models. The primary endpoint consisted of all-cause death, heart transplantation or implantation of ventricular assist device (VAD) (52 events after 12 months). The secondary outcome included sudden cardiac death, resuscitated cardiac arrest or treated ventricular tachyarrhythmia (47 events after 12 months, including recurrence in 6 subjects after 12 months).

| 1. **12-month univariate predictors of the primary outcome (all cause mortality, heart transplantation or implantation of VAD)** | | | | | | |
| --- | --- | --- | --- | --- | --- | --- |
| **Variable** | **Chi-square** | **p-value** | **Category for calculation of hazard ratios** | **Hazard ratio** | **95% CI** | **Pairwise comparison**  **(p-value)** |
| Sex | 6.86 | 0.009** | Males vs. females | 2.69 | 1.25-5.81 |  |
| Baseline history of persistent atrial fibrillation | 7.67 | 0.006** | Present vs. absent | 2.23 | 1.25-3.99 |  |
| Decompensated heart failure at baseline | 11.99 | 0.001** | Present vs. absent | 2.70 | 1.51-4.84 |  |
| NYHA class at 12 months | 16.75 | 0.000*** | Per class increase | 2.34 | 1.54-3.55 |  |
| LVEDD at 12 months (mm/m^2^) | 18.23 | 0.000*** | Per 10 unit increase | 2.54 | 1.64-3.92 |  |
| LVEF at 12 months (%) | 31.87 | 0.000*** | Per unit increase | 0.91 | 0.88-0.94 |  |
| LVEF ˃35% at 12 months | 32.01 | 0.000*** | Present vs. absent | 0.12 | 0.05-0.29 |  |
| LVRR at 12 months | 12.87 | 0.000*** | Present vs. absent | 0.29 | 0.14-0.59 |  |
| Restrictive mitral inflow pattern at 12 months  (n=293) | 21.58 | 0.000*** | Present vs. absent | 5.15 | 2.39-11.12 |  |
| E/E´ ratio at 12 months  (n=314) | 16.16 | 0.000*** | Per unit increase | 1.15 | 1.07-1.34 |  |
| RVD1 at 12 months  (mm) (n=199) | 22.32 | 0.000*** | Per unit increase | 1.22 | 1.13-1.33 |  |
| TAPSE et 12 months (mm) (n=309) | 8.80 | 0.003** | Per unit increase | 0.88 | 0.81-0.96 |  |
| Left atrium diameter at 12 months (mm)  (n=349) | 26.04 | 0.000*** | Per unit increase | 1.11 | 1.07-1.16 |  |
| LAVI at 12 months (ml/m^2^)  (n=98) | 17.75 | 0.000*** | Per unit increase | 1.08 | 1.04-1.13 |  |
| Mitral regurgitation at 12 months ≥ moderate | 29.73 | 0.000*** | Present vs. absent | 4.48 | 2.48-8.08 |  |
| B-type natriuretic peptide at 12 months  (n=121) | 69.87 | 0.000*** | Per 100 ng/l increase | 1.42 | 1.09-1.19 |  |
| VOIs class 4-5 in genes coding nuclear envelope | 4.63 | 0.031* | Positive vs. negative | 4.20 | 1.01-17.46 |  |
| **2. 12-month univariate predictors of the secondary outcome (sudden cardiac death, resuscitated cardiac arrest or treated ventricular tachyarrhythmia).** | | | | | | |
| **Variable** | **Chi-square** | **p-value** | **Category for calculation of hazard ratios** | **Hazard ratio** | **95% CI** | **Pairwise comparison**  **(p-value)** |
| Sustained ventricular tachycardia at baseline or during first 12 months (before 12m) | 20.49 | 0.000*** | Present vs. absent | 4.64 | 2.23-9.63 |  |
| Decompensated heart failure at baseline | 7.99 | 0.005** | Present vs. absent | 2.31 | 1.27-4.20 |  |
| LVEDD index at 12 months (mm/m^2^) | 19.89 | 0.000*** | Per 10 unit increase | 2.83 | 1.79-4.48 |  |
| LVEF at 12 months (%) | 24.24 | 0.000*** | Per unit increase | 0.93 | 0.90-0.96 |  |
| LVEF ˃35% at 12 months | 26.58 | 0.000*** | Present vs. absent | 0.18 | 0.09-0.38 |  |
| LVRR at 12 months | 14.38 | 0.000* | Present vs. absent | 0.27 | 0.13-0.56 |  |
| Restrictive mitral inflow pattern at 12 months (n=290) | 6.62 | 0.010* | Present vs. absent | 3.32 | 1.26-8.77 |  |
| RVD1 at 12 months (mm) (n=197) | 11.31 | 0.001** | Per unit increase | 1.14 | 1.06-1.24 |  |
| Left atrium diameter at 12 months (mm)  (n=345) | 11.42 | 0.001** | Per unit increase | 1.08 | 1.03-1.14 |  |
| LAVI (ml/m^2^) (n=96) | 11.71 | 0.001** | Per unit increase | 1.08 | 1.03-1.13 |  |
| Mitral regurgitation at 12m ≥ moderate (n=355) | 9.05 | 0.003** | Present vs. absent | 2.80 | 1.39-5.65 |  |
| B-type natriuretic peptide (ng/l) (n= 71) | 3.96 | 0.047* | Per 100 ng/l increase | 1.07 | 1.01-1.15 |  |
| Main categories of VOIs (negative, VUS, *TTNtv* class 4-5, non-titin class 4-5) | 7.88 | 0.049* | VUS vs. negative  Titin vs. negative  Non-titin vs. negative | 2.76  1.07  2.68 | 0.89-3.85  0.43-2.65  1.22-5.91 | 0.097  0.889  0.015* |
| Negative result of WES | 3.23 | 0.073 | Negative vs. others | 0.57 | 0.30-1.06 |  |
| VOIs class 4-5 in non-titin genes | 5.06 | 0.024* | Positive vs. negative | 2.13 | 1.08-4.20 |  |
| VOIs class 4-5 in genes coding nuclear envelope | 7.50 | 0.006** | Positive vs. negative | 5.80 | 1.39-24.14 |  |
| VOIs class 4-5 in genes coding cytoskeleton | 13.63 | 0.000*** | Positive vs. negative | 6.79 | 2.09-22.08 |  |
| **3. 12-month multivariate models predicting the primary outcome (all cause mortality, heart transplantation or implantation of VAD)**  **Model A)** sex, NYHA 12 months, LVEF 12 months, LVEDD index 12 months, VOIs class 4-5 in genes coding nuclear envelope  **Model B)** sex, NYHA 12 months, LVEF 12 months, E/E´ at 12 months, VOIs class 4-5 in genes coding nuclear envelope  **Model C)** sex, NYHA 12 months, LVRR 12 months, mitral regurgitation ≥ moderate, VOIs class 4-5 in genes coding nuclear envelope  **Model D)** sex, NYHA 12 months, LVRR 12 months, E/E´ at 12 months, VOIs class 4-5 in genes coding nuclear envelope | | | | | | |
|  | **Wald score** | **p-value** | **Category for calculation of hazard ratios** | **Hazard ratio** | **95% CI** | **Pairwise comparison**  **(p-value)** |
| **Model A)**  Sex | 5.97 | 0.015* | Males vs. females | 2.70 | 1.22-5.97 |  |
| LVEF at 12 months | 29.39 | 0.000*** | Per unit increase | 0.91 | 0.88-0.94 |  |
| **Model B)**  Sex | 6.09 | 0.014* | Males vs. females | 3.80 | 1.32-11.0 |  |
| NYHA class | 6.98 | 0.008** | Per class increase | 2.33 | 1.24-4.35 |  |
| LVEF at 12 months | 15.88 | 0.000*** | Per unit increase | 0.92 | 0.88-0.96 |  |
| **Model C)**  Sex | 9.70 | 0.002** | Males vs. females | 3.54 | 1.60-7.85 |  |
| NYHA class | 9.75 | 0.002** | Per class increase | 1.99 | 1.29-3.07 |  |
| Mitral regurgitation at 12 months ≥ moderate | 20.08 | 0.000*** | Present vs. absent | 4.13 | 2.22-7.67 |  |
| **Model D)**  Sex | 9.06 | 0.003** | Males vs. females | 5.18 | 1.77-15.13 |  |
| NYHA class | 10.04 | 0.002** | Per class increase | 2.59 | 1.44-4.67 |  |
| E/E´ ratio at 12 months | 21.17 | 0.001** | Per unit increase | 1.39 | 1.05-1.23 |  |
| **4. 12-month multivariate models predicting the secondary outcome (sudden cardiac death, resuscitated cardiac arrest and treated ventricular tachyarrhythmia)**  **Model A)** Sustained ventricular tachycardia before 12M**,** LVEF 12 months, LVEDD 12 months, main categories of VOIs (negative, VUS, *TTNtv* class 4-5, non-titin class 4-5)  **Model B)** Sustained ventricular tachycardia before 12M, LVEF 12 months, LVEDD 12 months, VOIs class 4-5 in non-titin genes  **Model C)** Sustained ventricular tachycardia before 12M, LVEF 12 months, LVEDD 12 months, VOIs class 4-5 in genes coding nuclear envelope  **Model D)** Sustained ventricular tachycardia before 12M, LVEF 12 months, LVEDD 12 months, VOIs class 4-5 in genes coding cytoskeleton  **Model E**) Sustained ventricular tachycardia before 12M, LVRR 12M, Mitral regurgitation at 12m ≥ moderate, main categories of VOIs (negative, VUS, *TTNtv* class 4-5, non-titin class 4-5)  **Model F)** Sustained ventricular tachycardia before 12M, LVRR 12M, Mitral regurgitation at 12m ≥ moderate, VOIs class 4-5 in non-titin genes  **Model G)** Sustained ventricular tachycardia before 12M, LVRR 12M, Mitral regurgitation at 12m ≥ moderate, VOIs class 4-5 in genes coding nuclear envelope  **Model H)** Sustained ventricular tachycardia before 12M, LVRR 12M, Mitral regurgitation at 12m ≥ moderate, VOIs class 4-5 in genes coding cytoskeleton | | | | | | |
|  | **Wald score** | **p-value** | **Category for calculation of hazard ratios** | **Hazard ratio** | **95% CI** | **Pairwise comparison**  **(p-value)** |
| **Model A)**  Sustained ventricular tachycardia before 12m | 11.18 | 0.001** | Present vs. absent | 3.52 | 1.68-7.37 |  |
| LVEF at 12 months (%) | 20.04 | 0.000*** | Per unit increase | 0.93 | 0.90-0.96 |  |
| **Model B)**  Sustained ventricular tachycardia before 12m | 11.18 | 0.001** | Present vs. absent | 3.52 | 1.68-7.37 |  |
| LVEF at 12 months (%) | 20.04 | 0.000*** | Per unit increase | 0.93 | 0.90-0.96 |  |
| **Model C)**  Sustained ventricular tachycardia before 12m | 10.56 | 0.001** | Present vs. absent | 3.41 | 1.63-7.15 |  |
| LVEF at 12 months (%) | 20.40 | 0.000*** | Per unit increase | 0.93 | 0.90-0.96 |  |
| VOIs class 4-5 in genes coding nuclear envelope | 5.16 | 0.023* | Positive vs. negative | 5.33 | 1.26-22.62 |  |
| **Model D)**  Sustained ventricular tachycardia before 12m | 11.18 | 0.001** | Present vs. absent | 3.52 | 1.68-7.37 |  |
| LVEF at 12 months (%) | 20.04 | 0.000*** | Per unit increase | 0.93 | 0.90-0.96 |  |
| **Model E)**  Sustained ventricular tachycardia before 12m | 13.47 | 0.000*** | Present vs. absent | 4.12 | 1.94-8.79 |  |
| LVRR at 12 months | 7.29 | 0.007** | Present vs. absent | 0.35 | 0.17-0.75 |  |
| Mitral regurgitation at 12 months ≥ moderate | 6.11 | 0.013* | Present vs. absent | 2.50 | 1.21-5.15 |  |
| **Model F)**  Sustained ventricular tachycardia before 12m | 13.47 | 0.000*** | Present vs. absent | 4.12 | 1.94-8.79 |  |
| LVRR at 12 months | 7.29 | 0.007** | Present vs. absent | 0.35 | 0.17-0.75 |  |
| Mitral regurgitation at 12 months ≥ moderate | 6.11 | 0.013* | Present vs. absent | 2.50 | 1.21-5.15 |  |
| **Model G)**  Sustained ventricular tachycardia before 12m | 11.18 | 0.001** | Present vs. absent | 3.75 | 1.73-8.14 |  |
| LVRR at 12 months | 7.61 | 0.006** | Present vs. absent | 0.34 | 0.16-0.74 |  |
| Mitral regurgitation at 12 months ≥ moderate | 5.43 | 0.020* | Present vs. absent | 2.38 | 1.15-4.95 |  |
| VOIs class 4-5 in genes coding nuclear envelope | 4.09 | 0.043* | Positive vs. negative | 4.72 | 1.05-21.24 |  |
| **Model H)**  Sustained ventricular tachycardia before 12m | 13.47 | 0.000*** | Present vs. absent | 4.12 | 1.94-8.79 |  |
| LVRR at 12 months | 7.29 | 0.007** | Present vs. absent | 0.35 | 0.17-0.75 |  |
| Mitral regurgitation at 12 months ≥ moderate | 6.11 | 0.013* | Present vs. absent | 2.50 | 1.21-5.15 |  |

**Abbreviations:** ACMG= American College of Medical Genetics and Genomics, BNP= B-type natriuretic peptide, LVEDD= left ventricular end-diastolic dimension, LVEF= left ventricular ejection fraction, LAVI= left atrial volume index, LVRR= left ventricular reverse remodeling, NT-proBNP= N-terminal pro-B-type natriuretic peptide, RVD1= basal right ventricular diameter, TAPSE= tricuspid annular systolic plane excursion, TTNtv = titin truncating variant, VOIs= variants of interest, WES = whole-exome sequencing.

**SUPPLEMENTAL REFERENCES:**

1. Pinto YM, Elliott PM, Arbustini E, Adler Y, Anastasakis A, Böhm M, et al. Proposal for a revised definition of dilated cardiomyopathy, hypokinetic non-dilated cardiomyopathy, and its implications for clinical practice: a position statement of the ESC Working Group on Myocardial and Pericardial Diseases. Eur Heart J 2016;37(23):1850-1858.
2. Ware JS, Amor-Salamanca A, Tayal U, Govind R, Serrano I, Salazar-Mendiguchía J et al. Genetic Etiology for Alcohol-Induced Cardiac Toxicity. J Am Coll Cardiol. 2018;71(20):2293-2302.
3. Huizar JF, Ellenbogen KA, Tan AY, Kaszala K. Arrhythmia-Induced Cardiomyopathy: JACC State-of-the-Art Review. J Am Coll Cardiol. 2019; 73(18): 2328–2344.
4. Gigli M, Merlo M, Graw SL, Barbati G, Rowland TJ, Slavov DB, et al. Genetic Risk of Arrhythmic Phenotypes in Patients With Dilated Cardiomyopathy. J Am Coll Cardiol 2019;74(11):1480-90.
5. Yancy CW, Jessup M, Bozkurt B, Butler J, Casey DE Jr, Drazner MH, et al. 2013 ACCF/AHA guideline for the management of heart failure: executive summary. Circulation 2013;128(16):1810-1852.
6. McKenna A, Hanna M, Banks E, Sivachenko A, Cibulskis K, Kernytsky A, et al. The Genome Analysis Toolkit: a MapReduce framework for analyzing next-generation DNA sequencing data. Genome Res 2010; 20: 1297–1303.
7. Cingolani P, Platts A, Wang le L, Coon M, Nguyen T, Wang L, et al. A program for annotating and predicting the effects of single nucleotide polymorphisms, SnpEff: SNPs in the genome of Drosophila melanogaster strain w1118; iso-2; iso-3. Fly (Austin) 2012;6:80-92.
8. Paila U, Chapman BA, Kirchner R, Quinlan AR. GEMINI: integrative exploration of genetic variation and genome annotations. PLoS Comput Biol 2013;9:e1003153.
9. Lek M, Karczewski KJ, Minikel EV, Samocha KE, Banks E, Fennell T, et al. Exome Aggregation Consortium. (2016). Analysis of protein-coding genetic variation in 60,706 humans. Nature, 536(7616):285-291.
10. Thorvaldsdóttir H, Robinson JT, Mesirov JP. Integrative Genomics Viewer (IGV): high-performance genomics data visualization and exploration. Brief Bioinformatics 2013;14:178-192.
11. Schwarz JM, Cooper DN, Schuelke M, Seelow D. MutationTaster2: mutation prediction for the deep-sequencing age. Nat Methods 2014;11(4):361-362
12. Peduzzi P, Concato J, Feinstein AR, Holford TR. Importance of events per independent variable in proportional hazards regression analysis. II. Acuracy and precision of regression estimates. J Clin Epidemiol 1995; 48:1503-10.
13. Bouzková K, Kubánek M, Krebsová A, Piherová L, Ridzoň P, Roubíček T, et al. Fukutinopathy as a rare cause of dilated cardiomyopathy and subclinical skeletal myopathy - a case report and review of cardiac involvement in skeletal muscle disease. Cor et Vasa 2022, 64(4):468-473.
14. Kubánek M, Schimerová T, Piherová L, Brodehl A, Krebsová A, Ratnavadivel S, et al. Desminopathy: Novel Desmin Variants, a New Cardiac Phenotype, and Further Evidence for Secondary Mitochondrial Dysfunction. J Clin Med. 2020; 9(4):937.
15. Majer F, Kousal B, Dusek P, Piherova L, Reboun M, Mihalova R, et al. Alu-mediated Xq24 deletion encompassing CUL4B, LAMP2, ATP1B4, TMEM255A, and ZBTB33 genes causes Danon disease in a female patient. Am J Med Genet A. 2020; 182(1):219-223.
16. Pannone L, Bocchinfuso G, Flex E, Rossi C, Baldassarre G, Lissewski C, et. al. Structural, Functional, and Clinical Characterization of a Novel PTPN11 Mutation Cluster Underlying Noonan Syndrome. Hum Mutat 2017; 38(4):451-459.
